# The Impact of Nonpharmacological Interventions on Patient Experience, Opioid Use, and Health Care Utilization in Adult Cardiac Surgery Patients: Protocol for a Mixed Methods Study

**DOI:** 10.2196/21350

**Published:** 2021-02-16

**Authors:** Alexander A Brescia, Julie R Piazza, Jessica N Jenkins, Lindsay K Heering, Alexander J Ivacko, James C Piazza, Molly C Dwyer-White, Stefanie L Peters, Jesus Cepero, Bailey H Brown, Faraz N Longi, Katelyn P Monaghan, Frederick W Bauer, Varun G Kathawate, Sara M Jafri, Melissa C Webster, Amanda M Kasperek, Nickole L Garvey, Claudia Schwenzer, Xiaoting Wu, Kiran H Lagisetty, Nicholas H Osborne, Jennifer F Waljee, Michelle Riba, Donald S Likosky, Mary E Byrnes, G Michael Deeb

**Affiliations:** 1 Department of Cardiac Surgery Michigan Medicine University of Michigan Ann Arbor, MI United States; 2 Center for Healthcare Outcomes and Policy University of Michigan Ann Arbor, MI United States; 3 Office of Patient Experience Michigan Medicine University of Michigan Ann Arbor, MI United States; 4 Department of Child and Family Life CS Mott Children's Hospital Michigan Medicine Ann Arbor, MI United States; 5 Frankel Cardiovascular Center Michigan Medicine Ann Arbor, MI United States; 6 Children and Women's Hospital Michigan Medicine Ann Arbor, MI United States; 7 Department of Psychiatry Michigan Medicine University of Michigan Ann Arbor, MI United States; 8 Department of Surgery Michigan Medicine University of Michigan Ann Arbor, MI United States

**Keywords:** cardiac surgery, patient experience, nonpharmacological interventions, child life specialists, opioids, anxiety, stress, depression

## Abstract

**Background:**

Despite pharmacological treatments, patients undergoing cardiac surgery experience severe anxiety and pain, which adversely affect outcomes. Previous work examining pediatric and nonsurgical adult patients has documented the effectiveness of inexpensive, nonpharmacological techniques to reduce anxiety and pain as well as health care costs and length of hospitalization. However, the impact of nonpharmacological interventions administered by a dedicated *comfort coach* has not been evaluated in an adult surgical setting.

**Objective:**

This trial aims to assess whether nonpharmacological interventions administered by a trained *comfort coach* affect patient experience, opioid use, and health care utilization compared with usual care in adult cardiac surgery patients. This study has 3 specific aims: assess the effect of a *comfort coach* on patient experience, measure differences in inpatient and outpatient opioid use and postoperative health care utilization, and qualitatively evaluate the *comfort coach* intervention.

**Methods:**

To address these aims, we will perform a prospective, randomized controlled trial of 154 adult cardiac surgery patients at Michigan Medicine. Opioid-naive patients undergoing first-time, elective cardiac surgery via sternotomy will be randomized to undergo targeted interventions from a *comfort coach* (intervention) versus usual care (control). The individualized *comfort coach* interventions will be administered at 6 points: preoperative outpatient clinic, preoperative care unit on the day of surgery, extubation, chest tube removal, hospital discharge, and 30-day clinic follow-up. To address aim 1, we will examine the effect of a *comfort coach* on perioperative anxiety, self-reported pain, functional status, and patient satisfaction through validated surveys administered at preoperative outpatient clinic, discharge, 30-day follow-up, and 90-day follow-up. For aim 2, we will record inpatient opioid use and collect postdischarge opioid use and pain-related outcomes through an 11-item questionnaire administered at the 30-day follow-up. Hospital length of stay, readmission, number of days in an extended care facility, emergency room, urgent care, and an unplanned doctor’s office visit will be recorded as the primary composite endpoint defined as total days spent at home within the first 30 days after surgery. For aim 3, we will perform semistructured interviews with patients in the intervention arm to understand the *comfort coach* intervention through a thematic analysis.

**Results:**

This trial, funded by Blue Cross Blue Shield of Michigan Foundation in 2019, is presently enrolling patients with anticipated manuscript submissions from our primary aims targeted for the end of 2020.

**Conclusions:**

Data generated from this mixed methods study will highlight effective nonpharmacological techniques and support a multidisciplinary approach to perioperative care during the adult cardiac surgery patient experience. This study’s findings may serve as the foundation for a subsequent multicenter trial and broader dissemination of these techniques to other types of surgery.

**Trial Registration:**

ClinicalTrials.gov NCT04051021; https://clinicaltrials.gov/ct2/show/NCT04051021

**International Registered Report Identifier (IRRID):**

DERR1-10.2196/21350

## Introduction

### Nonpharmacological Interventions

Adults undergoing inpatient surgery commonly develop both severe perioperative anxiety [1-5] (28%-70% of patients) and postoperative pain (54%-74% with moderate-to-severe pain at discharge), [6,7] both of which may significantly affect patient outcomes [8-11]. Nonpharmacological interventions such as distraction techniques, guided imagery, music, and art have been found to be inexpensive complements to pharmacologic treatments, effectively reducing both anxiety and acute and chronic pain [12-14] in pediatric [15] and hospitalized nonsurgical adult patients [16-19] when administered routinely by a dedicated child life specialist *comfort coach*. However, the impact of nonpharmacological interventions and the role of a comfort coach in an adult surgical setting has not been evaluated.

### Certified Child Life Specialists

Certified child life specialists are frontline health care professionals trained to provide psychosocial care to pediatric patients and families facing stressful medical experiences. Child life specialists conduct thorough assessments and build therapeutic relationships with patients and families to support them to cope and protect emotional safety. Individualized interventions include fostering healing environments, therapeutic play, nonpharmacological pain management, procedural preparation, diagnosis teaching, sibling support, medical play, and bereavement support. Child life specialists obtain a bachelor’s degree with an emphasis on psychology, education, and human development. They complete comprehensive clinical training, pass a certification exam, and maintain certification through targeted continuing education [20].

### Applications in the Pediatric Setting and Translation to the Adult Setting

Child life specialists work with pediatric patients to teach individualized coping strategies to mitigate anxiety and manage both acute and chronic pain. Play is the universal language of all children, and play-based coping strategies foster expression and promote a sense of control and mastery. Nonpharmacological interventions effective in pediatric and adolescent patients include calm breathing techniques, distraction, guided imagery, art, muscle relaxation, music, environmental modification, and comfort positioning [20]. The child life specialist’s role has resulted in reduced pain, anxiety, use of analgesics, and length of stay [16]. We often assume that adults can handle medical stressors and employ coping skills, but many adults do not have such skills established [21]. In addition, this has resulted in underestimating adult patients’ pain effects [22] and often not addressing pain with known effective nonpharmacological interventions in combination with pain medication [13]. Building on the effectiveness and essential skills that have been dedicated to pediatric patients can facilitate opportunities for adult patients to reframe health care experiences and provide coaching through pain and anxiety, adding to their coping skills to support comfort. Applying child life strategies across the age span can further enhance the human experience of health care [23].

### Conceptual Model

**Figure 1:**
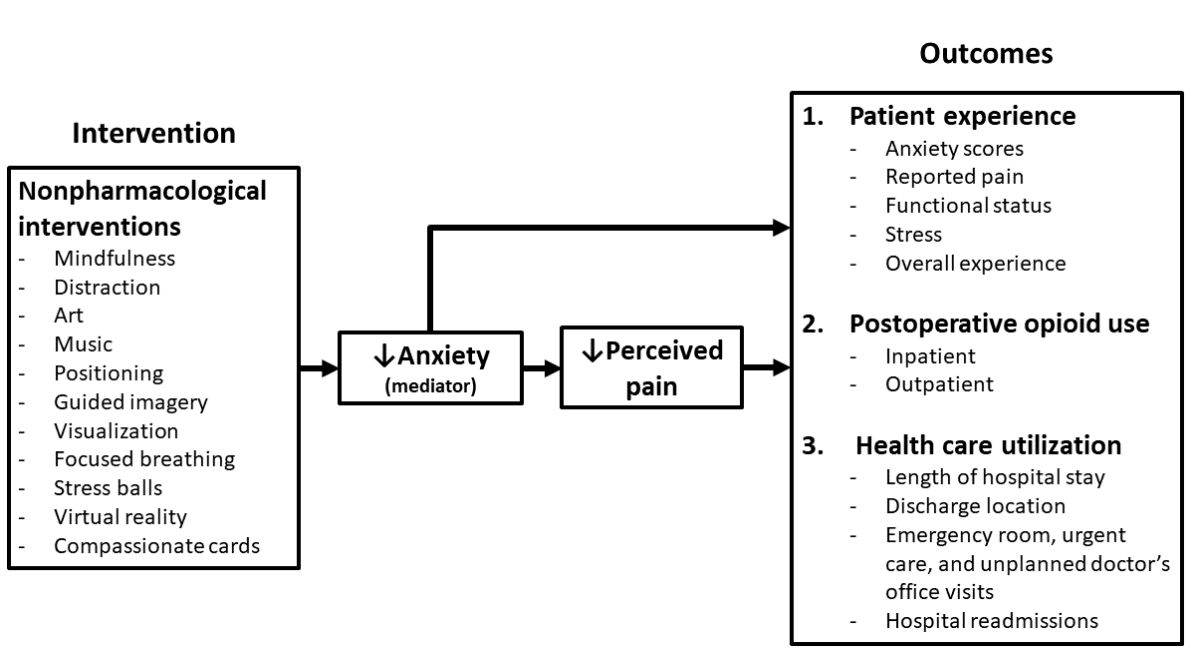
Conceptual model. This conceptual model demonstrates the hypothesized effect of nonpharmacological interventions on the outcomes in this study rooted in gate-control theory.

We propose that providing a dedicated, trained comfort coach administering nonpharmacological interventions will improve cardiac surgery outcomes, including anxiety, self-reported pain, opioid use, and health care utilization. Periprocedural nonpharmacological approaches are well established in pediatrics [24,25], especially for needle-related pain [26]. Nonpharmacological approaches to acute pain management are rooted in the *gate-control theory* [27,28]. This theory suggests that descending nerve impulses from the brain, including thoughts, beliefs, emotions, and attention, can influence the ascending pain signal from tissue damage. In the context of surgery, anxiety may heighten pain, whereas attention focused on pleasant activity might decrease pain. In this context, many nonpharmacological interventions mitigate anxiety, which mediates pain perception, with both decreased anxiety and perception of pain affecting patient outcomes (Figure 1).

### Rationale for the Study

Approximately 300,000 adults undergo cardiac surgery in the United States each year [29], with 90-day episode payments combined to exceed US $15 billion [30-32]. Cardiac surgery through a sternotomy has been shown to cause severe anxiety and pain that persist in spite of pharmacologic pain control with opioids [5,33,34], with up to 81% reporting some pain at 1 month [35] and up to 43% reporting persistent pain at 1 year [36]. Rates of persistent opioid use and long-term opioid dependence in cardiac surgery patients have increased 8-fold over the past 15 years, resulting in higher postoperative complication rates, prolonged hospital length of stay, and increased health care costs [37]. Moreover, opioid prescription sizes remain high, and overprescribing is common [38-47]. Whereas assessments of individual nonpharmacological interventions such as preoperative education [48,49], massage [50], and music [34] have demonstrated an improvement in self-reported pain scores after cardiac surgery, differences in opioid use have not been reported. Furthermore, individualized nonpharmacological interventions by a comfort coach have been shown to reduce the length of hospital stay [15,51] and health care costs [52,53] in pediatric patients, prompting the establishment of child life services as a *quality benchmark* and *indicator of excellence in pediatric care*, [15,54]. In contrast, these techniques have not been studied in adult cardiac surgery patients.

Data generated by this study may highlight effective techniques and support a multidisciplinary approach to nonpharmacological interventions to decrease pain, opioid use, anxiety, and health care utilization while increasing patient comfort and overall satisfaction during the adult cardiac surgery patient experience.

## Methods

### Overall Study Design

We will perform a prospective, double-armed, randomized, controlled trial of 154 cardiac surgical patients at a large academic center to assess whether nonpharmacological interventions by a trained comfort coach affect patient experience, opioid use, and health care utilization as compared with usual care. The individualized comfort coach interventions will be administered at 6 time-points: (1) at the preoperative clinic, (2) on the day of surgery, (3) at extubation, (4) at chest tube removal, (5) at hospital discharge, and (6) at the 30-day clinic follow-up.

This study has 3 specific aims: (1) assess the effect of a comfort coach on patient experience, (2) measure differences in inpatient and outpatient opioid use and postsurgical health care utilization, and (3) qualitatively evaluate the effectiveness of the comfort coach intervention. Toward aim 1, we will use validated survey metrics to capture anxiety and depression (preoperative clinic, discharge, 30-day follow-up, and 90-day follow-up), functional status (preoperative clinic and 30-day follow-up), surgery-related psychological stress (30-day follow-up), patient-reported in-hospital pain (discharge), and patient experience (30-day follow-up). Toward aim 2, we will compare inpatient and postdischarge opioid use and patient-reported outcomes, including pain scores and pain management practices between groups. In addition, the composite primary endpoint of the study will be recorded as the total number of days spent at home out of the first 30 after surgery, incorporating hospital length of stay, readmissions, number of days in an extended care facility, emergency room, urgent care, and unplanned doctor visits. Aim 3 will include semistructured interviews of patients in the intervention group to understand the role, impact, and acceptability of a comfort coach.

All requirements for conducting human subjects research at the University of Michigan have been met. The study protocol has been reviewed, and the University of Michigan Institutional Review Board has approved this trial (quantitative, HUM00161399, and qualitative, HUM00170502). This clinical trial has been registered at ClinicalTrials.gov (NCT04051021).

### Patient Enrollment and Randomization

Cardiac surgical patients will be recruited from the Frankel Cardiovascular Center (FCVC) at Michigan Medicine. Opioid-naive patients undergoing first-time, elective cardiac surgery through a full median or miniature sternotomy beginning September 3, 2019, were screened for a targeted enrollment of 154 patients for randomization. Approximately 600 to 750 patients meeting these criteria underwent surgery at Michigan Medicine in 2017. Opioid-naive is defined as opioid-free at the time of preoperative clinic history and physical examination.

Patients lost to follow-up will be censored for data analysis, and missing individual intervention points will be dealt with by multiple imputations, as appropriate. We will compare the clinical and sociodemographic attributes of patients who decline participation and patients lost to follow-up with those of patients with complete follow-up to assess potential responder bias. We estimated a 20% rate of missingness, which was incorporated into our power calculation. On the basis of historical institutional data, we anticipated approaching 5 to 10 patients per week for potential enrollment, with an approximate 5 to 7 month enrollment period within a 12-month study period for completion of clinical follow-up, data analysis, and manuscript production. Although this timeline was affected by the COVID-19 pandemic forcing a pause in enrollment between March 14th and the time of writing, we were 93.5% (144/154) enrolled at the time of pausing and remained optimistic about completing enrollment within our 12-month study period. We used block randomization with randomly variable block sizes generated with Stata 15 software (StataCorp LLC, College Station, TX) and computer randomization occurred with the treatment assignment tool (Treatment Assignment Tool-University of Michigan [TATUM]) from the Michigan Institute for Clinical and Health Research.

**Figure 2:**
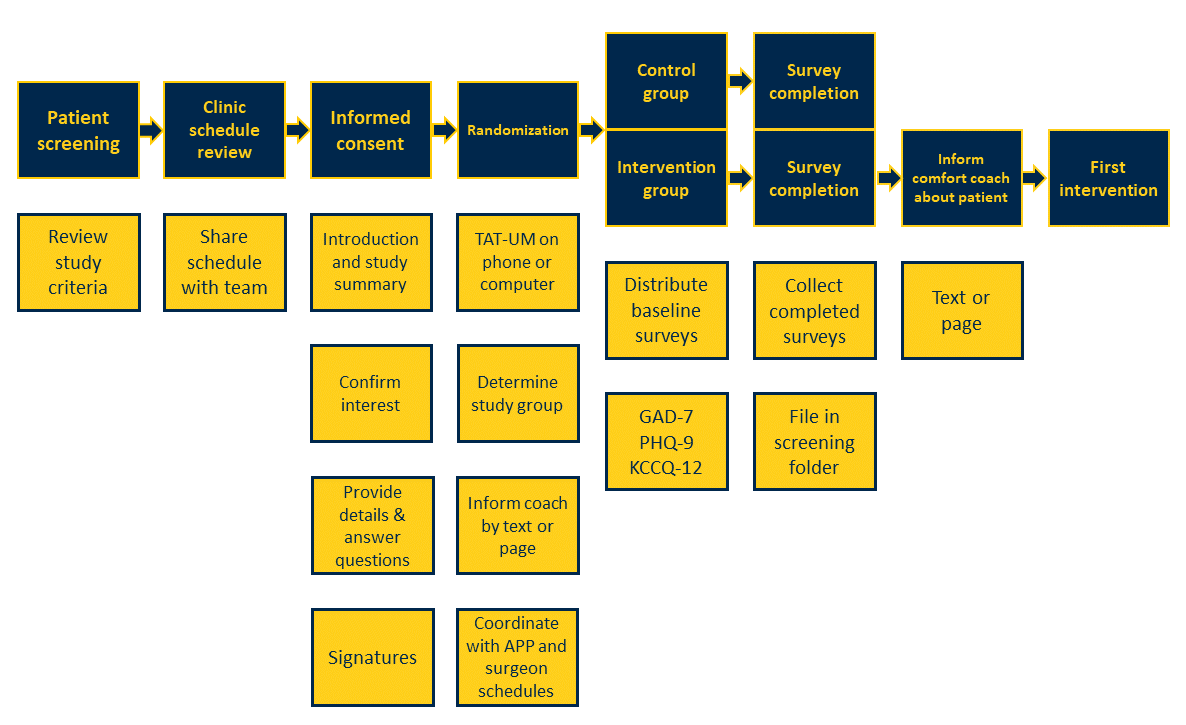
Preoperative clinic value stream map. Blue blocks with maize writing indicate the main steps in the clinic process, whereas maize blocks with blue writing summarize tasks performed by the study coordinator. APP: advanced practice providers; GAD-7: Generalized Anxiety Disorder 7-item scale; KCCQ-12: Kansas City Cardiomyopathy Questionnaire 12-item short-form; PHQ-9: Patient Health Questionnaire-9; TATUM: Treatment Assignment Tool-University of Michigan.

Patients were approached, participated in the informed consent process, and were enrolled during the preoperative history and physical appointment, typically after being seen by a member of the advanced practice team. The study coordinator approached potential subjects either in exam rooms or in the clinic waiting room to describe the study and offer participation. Interested patients engaged with the study coordinator and gave informed consent, at which point the coordinator randomized and assigned a sequential subject identification number to each study patient using TATUM (Figure 2).

### The Comfort Coach Approach and Interventions

In this trial, the *comfort coach* is a trained Certified Child Life Specialist who provides therapeutic interventions that have been shown to reduce anxiety and pain medication use while increasing patient and family satisfaction [15]. Child life specialists use evidence-based nonpharmacological pain management techniques such as preparing patients for painful encounters, comforting and reassuring them, coping strategies such as distraction, and offering positive reinforcement to support patients undergoing painful procedures [55]. These interventions focus on improving patient experience, validating emotions, and offering an individualized approach with the goal of reducing pain and anxiety. Focused breathing and guided imagery with targeted relaxation and pain management outcomes will support patient choice and engagement to encourage sustainable coping skills.

Child life specialists are trained in lifespan development and family systems theories, specifically addressing pain and anxiety through individualized comfort techniques. Though they are trained specifically in pediatrics, these coping strategies translate across ages. Adults routinely express the desire for distraction techniques, guided imagery, and preparation procedures during procedural care [16,23]. Moreover, adults may benefit, similar to pediatric patients, from nonpharmacological interventions when undergoing cardiac surgery. Whereas adults may have more experience with anxiety and pain management than children, new perioperative experiences have the potential to raise anxiety levels that can be mitigated by simple comfort strategies. Thus, child life specialists are ideally equipped to address perioperative anxiety and painful situations in both children and adults. We performed an 11-patient feasibility study in patients who underwent open cardiac surgery at Michigan Medicine using certified child life specialists, which demonstrated that nonpharmacological comfort coach interventions were feasible.

In this study, patients randomized to the intervention group will meet the comfort coach in the preoperative clinic (#1), where the coach will perform an introductory emotional, medical, and psychosocial needs assessment to consider the impact of surgery on the patient. The comfort coach will also introduce a *comfort menu* (Multimedia Appendix 1) [56-63] for pain management. After the preoperative clinic visit, the comfort coach will see the patient 5 additional times: at the preoperative bay immediately before surgery (#2), at extubation in the intensive care unit (**#**3), at chest tube removal (#4), at discharge (#5), and at the 30-day clinic follow-up visit (#6). These points of intervention were defined through feedback provided during our initial 11-patient feasibility study. During these intervention points, patients will receive the comfort menu and choose individualized interventions, including guided imagery, guided visualization, focused breathing, distraction techniques, music, art, mindfulness, and patient positioning. Supplies used during comfort coach interventions commonly include music playlists, compassion cards, stress balls, iPad activities, and virtual reality goggles. Additional activities are listed in the comfort menu in Multimedia Appendix 1.

### Study Overview and Pretrial CONSORT (Consolidated Standards of Reporting Trials) Flow Diagram

**Figure 3:**
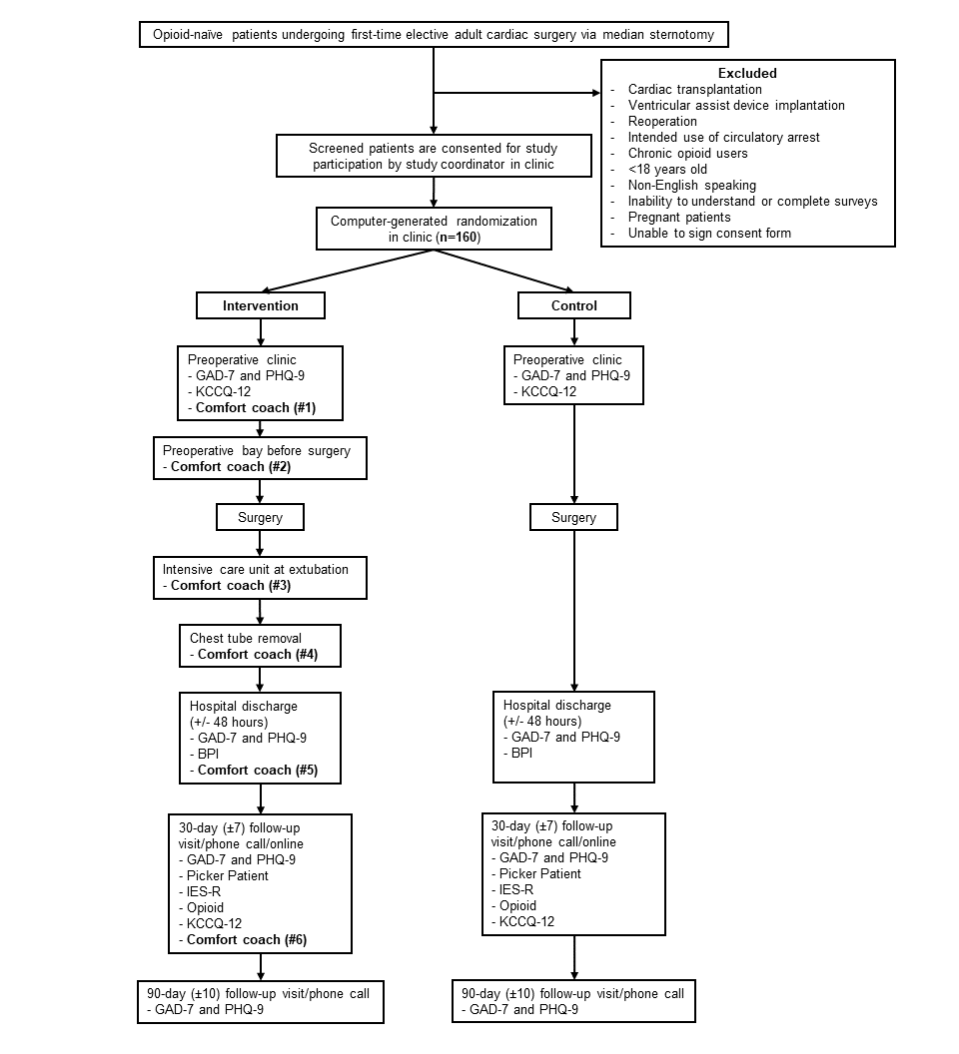
Clinical trial CONSORT (Consolidated Standards of Reporting Trials) diagram. Patient population and clinical trial flow diagram. The timing of each *comfort coach* intervention touchpoint is indicated, and each touchpoint is numbered. BPI: Brief Pain Inventory; GAD-7: Generalized Anxiety Disorder 7-item scale; IES-R: Impact of Events Scale-Revised; KCCQ-12: Kansas City Cardiomyopathy Questionnaire 12-item short-form; PHQ-9: Patient Health Questionnaire-9.

Screening, enrollment, randomization, and study design are summarized in Figure 3.

#### Aim 1: Assess the Effect of a Comfort Coach on Patient Experience

To address this aim, we will examine the effect of the comfort coach on perioperative anxiety, self-reported pain, functional status, and patient satisfaction. We will use the Generalized Anxiety Disorder 7-item scale (GAD-7) [56] anxiety assessment and Patient Health Questionnaire-9 (PHQ-9) [57] depression scale at 4 points: preoperative clinic visit, discharge, and at 30- and 90-day follow-up. The Kansas City Cardiomyopathy Questionnaire short-form (KCCQ-12) [58,59] will be administered at the preoperative clinic visit and 30-day follow-up to evaluate functional status. The Impact of Events Scale-Revised (IES-R) [60] will measure event-related psychological stress at 30-day follow-up, and the Picker Patient Experience Questionnaire [61] will be administered at the 30-day follow-up. The Brief Pain Inventory [62,63] will be administered at discharge to assess in-hospital pain. We hypothesize that patients who receive a comfort coach report a better hospital experience, improved functional recovery, and lower levels of depression, anxiety, stress, and pain.

##### Outcome Measures for Aim 1

Outcome measures for aim 1 exclusively consist of validated measures for anxiety, depression, stress, pain, and patient satisfaction (Textbox 1). These validated tools were adapted to better evaluate differences in pharmacological and nonpharmacological treatments and their corresponding effects and are included in the Multimedia Appendix 1.

**Evaluative surveys and questionnaires.**

Generalized Anxiety Disorder 7-item Scale

- A 7-item validated questionnaire to assess and potentially diagnose generalized anxiety disorder [56].

Patient Health Questionnaire

- A 9-item validated questionnaire which generates a total score out of 27 used to diagnose 5 different degrees of depressive disorders [57].

Impact of Events Scale-Revised

- A 22-item validated scale used to measure event-related stress with the potential to indicate clinical suspicion or diagnosis of posttraumatic stress disorder [60].

Picker Patient Experience Questionnaire

- A 15-item questionnaire designed to capture the patient’s inpatient experience [61].

Brief Pain Inventory

- Short-form 9-question inventory assessing patient pain location, severity, relief, and activity level [62,63].

Postoperative Opioid and Pain Management Questionnaire

- An 11-item questionnaire developed at Michigan Medicine for collecting data on opioids prescribed, opioids used, pain scores, opioid storage and disposal practices, and assessment of opioid education.

Kansas City Cardiomyopathy Questionnaire

- A 12-item questionnaire assessing the impact of heart failure on the patient’s daily activities and lifestyle [58,59].

The survey and questionnaire instruments were used as part of specific aim #1 to evaluate anxiety (Generalized Anxiety Disorder 7-item scale [GAD-7]), depression (PHQ-9), functional status (Kansas City Cardiomyopathy Questionnaire short-form [KCCQ-12]), stress (Impact of Events Scale-Revised [IES-R]), and patient experience (Picker Patient). Patient-reported pain levels (BPI) and postdischarge opioid use and pain management practices (Postoperative Opioid and Pain Management Questionnaire [OPIOID]) were collected as part of specific aim #2.

##### Analytic Approach

Mean total GAD-7 and PHQ-9 scores at each of the 4 time-points (Table 1) will be compared between the 2 groups with two-tailed *t* tests, whereas categorical findings (eg, mild, moderate, or severe anxiety on the GAD-7 and minimal, mild, moderate, moderately severe, or severe depression on the PHQ-9) will be compared between groups using chi-square tests. In addition, repeated measures ANOVA (analysis of variance) tests will be performed for the GAD-7 and PHQ-9 across the 4 time-points to examine differences between groups while accounting for correlation over time within each patient. IES-R mean total scores will be compared between groups using a two-tailed *t* test, and the proportion of patients in each group meeting established cutoffs (≥24, ≥33, and ≥37) [64-66] will be compared with chi-square or Fisher exact tests, as appropriate. Chi-square tests will be used for the Picker Patient Experience Questionnaire to determine differences in mean proportions of *problems* reported out of the 15 items in the survey. The mean intrapatient difference between preoperative clinic and 30-day follow-up in functional status scores on the KCCQ-12 will be evaluated (≥5 point difference indicates a clinically important difference) and compared between study cohorts. Pain severity and pain interference scores will be determined (both out of 10) from the Brief Pain Inventory and generated for each patient. These 2 mean scores will be compared between groups using a two-tailed *t* test. Finally, pain score proportions on a 4-point scale (none, minimal, moderate, or severe) recorded through the 30-day postoperative questionnaire will be compared across groups using the chi-square test.

**Table 1:** Schedule of survey instrument administration. The survey administration schedule is indicated for both study cohorts (ie, all patients).

Preoperative clinic visit for History and Physical	Day of discharge	30-day postoperative clinic visit	90-day postoperative clinic visit or phone call
GAD-7^a^	GAD-7	GAD-7	GAD-7
PHQ-9^b^	PHQ-9	PHQ-9	PHQ-9
KCCQ-12^c^	BPI^d^	Picker Patient^e^	N/A^f^
N/A	N/A	IES-R^g^	N/A
N/A	N/A	OPIOID^h^	N/A
N/A	N/A	KCCQ-12	N/A

^a^GAD-7: Generalized Anxiety Disorder 7-item scale.

^b^PHQ-9: Patient Health Questionnaire-9.

^c^KCCQ-12: Kansas City Cardiomyopathy Questionnaire 12-item short-form.

^d^BPI: Brief Pain Inventory.

^e^Picker Patient: Picker Patient Experience Questionnaire.

^f^N/A: not applicable.

^g^IES-R: Impact of Events Scale-Revised.

^h^OPIOID: Postoperative Opioid and Pain Management Questionnaire.

#### Aim 2: Measure Differences in Inpatient and Outpatient Opioid Use and Postsurgical Health Care Utilization

Inpatient postoperative opioid use will be recorded for 3 inpatient calendar days before discharge in oral morphine equivalents (OME) per day. Outpatient opioid use and pain scores will be assessed through an 11-item questionnaire administered at 1-month follow-up. Hospital length of stay, number of days in an extended care facility, emergency room, urgent care, unplanned doctor office visits, and readmission will be recorded in a composite endpoint defined as total days spent at home within the first 30 days after surgery. We hypothesize that patients who receive a comfort coach consume less opioids after surgery and demonstrate lower postsurgical health care utilization.

##### Outcomes Measures for Aim 2

###### Opioid Use

Opioid amounts will be converted to OME [67]. Inpatient opioid use will be obtained through electronic chart review for the 3 inpatient calendar days before discharge (intravenous and oral) and will be reported daily in OME. Outpatient opioid use will be collected using the 11-item questionnaire administered at the 30-day postoperative clinic appointment and will be reported as total OME consumed postdischarge. Additional data captured through chart reviews and clinic questionnaires include the type of opioid, the amount prescribed (OME), number of refills, outpatient storage location, and opioid education received regarding risks and proper opioid disposal.

###### Postsurgical Health Care Utilization

The primary endpoint of this trial is a composite outcome defined as the total number of days spent at home within the first 30 days after surgery. Each partial or full day spent in the hospital (during index or readmission hospitalization), at any extended care facility, emergency room, urgent care center, or doctor’s office for an unplanned visit will be subtracted from 30 to generate the total number of full days spent at home. This number will reflect total health care utilization within the immediate postoperative period, with lower values indicating more utilization. The number of days in an extended care facility, outside hospital emergency room or urgent care visits, and readmissions are routinely discussed at the 30-day postoperative clinic appointment and will be captured through a combination of chart review by 2 study team members and conversations with patients. In addition, the number of telephone calls made by each patient or patient’s family member to the University of Michigan hospital system regarding the clinical concerns of the patient within the first 30 postoperative days will be captured through chart review and independently verified by 2 study team members. If necessary, we will then use Michigan Value Collaborative data to quantify differences in health care utilization by comparing total and component 90-day episode payments among Medicare, Blue Cross Blue Shield of Michigan, and Medicaid beneficiaries, as our team has previously done in both coronary artery bypass (CABG) [31] and aortic valve replacement [30] studies in Michigan.

###### Analytic Approach

Mean inpatient opioid use, prescription size, postdischarge opioid use (in OME), and mean postsurgical health care utilization days will all be compared between groups using two-tailed *t* tests. Individual health care utilization outcomes will also be compared separately with two-tailed *t* tests for continuous data and chi-square tests for categorical data.

#### Aim 3: Qualitatively Evaluate the Effectiveness of the Comfort Coach Intervention

We will perform semistructured one-on-one interviews with 50 patients who had a comfort coach to understand (1) their experience with and perceived role of the intervention on their surgical experience and (2) the acceptability of the intervention. Insights from this thematic analysis will guide the identification and development of tools for broader implementation.

##### Semistructured Interviews

Interviews will be conducted by 2 study members (AB and MB) either in the FCVC cardiac surgery outpatient clinic area or over the telephone. Interviews will be audio recorded on an encrypted recorder, transcribed verbatim by an external HIPAA (Health Insurance Portability and Accountability Act)–approved professional transcriptionist, and redacted for all identifying information. Participants will be compensated with a gift card for their participation. After March 14, 2020, interviews were exclusively performed over the phone because of human subject research restrictions owing to the COVID-19 pandemic.

##### Analytic Approach

An initial interview guide will be developed and modified during the interview period through iterative steps. Data will be coded in MaxQDA20 (VERBI Software, 2019) qualitative analysis software. The team will meet to examine codes and identify emerging patterns and concepts that will be organized into themes. We will use the thematic analysis framework [68] to identify themes among patients who received a comfort coach and categorize these themes into 3 broad categories: (1) the role of the coach, (2) the impact of the coach, and (3) the acceptability of coaching. The data generated from these interviews will inform subsequent refining of our coaching intervention and development of tools for broader implementation of the intervention.

##### Power Analysis

Our primary endpoint is composite of health care utilization, defined as the total number of days spent at home within the first 30 days after surgery. Secondary endpoints include mean GAD-7 and PHQ-9, KCCQ-12, Picker Patient Experience Questionnaire, IES-R, and Brief Pain Inventory Scores and mean inpatient and outpatient opioid use.

We will compare mean days with a two-tailed *t* test and set the power of this study at 80% with alpha (Type I error) of .05 and the Cohen *d* effect size (defined as the difference in means divided by standard deviation) between medium (0.5) and large (0.8) [69,70], which yields a minimum sample size of 52 patients to detect a large effect size and 128 to detect a medium effect size [71]. Incorporating 20% missingness, a sample size of 154 patients will adequately detect a medium effect size. We next verified this statistically derived sample size with clinically relevant examples. Although most large series reporting opioid use report the median and interquartile range of OME [47,72], our sample size should be sufficient to satisfy the central limit theorem and use mean values. At our institution, the mean opioid use after sternotomy in 2017 was 200 OMEs. We estimated that to detect a 30% reduction in opioid use (mean 140 OMEs) with a standard deviation of 125 OME in the control and 100 OME in the intervention arms, a sample size of 114 would be required. For composite health care utilization, demonstrating a 3-day difference between the arms with a 6-day standard deviation would detect by a total sample size of 128 patients. With 154 patients, we should have adequate power to detect a difference in our primary outcomes for aims 1 and 2.

## Results

### Preliminary Data

An 11-patient feasibility study in aortic surgery patients (HUM00138828) was performed at the FCVC, 6 of whom were randomized to the control and 5 to the intervention arm. Extremely useful insight from this feasibility trial included feedback from intervention patients regarding the time-points at which their dedicated, trained comfort coach was most beneficial. This feedback was used to solidify the 6 touchpoints for our full clinical trial. In addition, an assumption about utilizing nonpharmacological interventions was that pain management and recovery after surgery were individualized processes. In contrast, we received feedback that family and relatives are intimately involved in patients' healing and emotional well-being. Furthermore, some patients indicated that their family members benefited from the comfort coach interventions, in some instances, even more than the patient. In addition, family interaction and socialization during the perioperative process were identified as important to pain management.

Executing the feasibility trial also provided direct insights for our study team. Using certified child life specialists in dual roles as full-time specialists at the children’s hospital and on-call for the feasibility trial proved to be a barrier to effective intervention, primarily because of the distance between the hospitals creating time-related challenges for meeting each touchpoint. Accordingly, these experiences informed the development of the full clinical trial by highlighting the importance of hiring a full-time, dedicated comfort coach to enhance the number of touchpoints met and increase care continuity. Most importantly, this preliminary trial demonstrated that it would be feasible to perform the comfort coach study protocol at the University of Michigan.

After completing the feasibility study, the study team performed telephone interviews of study patients and former open-heart surgery patients through the FCVC’s Patient Family Advisory Council to gain further insight into the patient experience and use this input to select the most appropriate survey instruments for the full clinical trial.

### Full Clinical Trial

Our clinical trial is funded by Blue Cross Blue Shield of Michigan Foundation, and enrollment is currently ongoing. As of June 2020, 144 patients have been enrolled and randomized in the trial, and 50 semistructured qualitative interviews have been performed. Since March 14, 2020, all survey touchpoints and interviews have been conducted remotely via telephone, online, or mail because of human subject research restrictions implemented at the University of Michigan to limit exposure to patients and staff during the COVID-19 pandemic. Comfort coach touchpoints for patients in the intervention group have continued during their inpatient hospitalization, whereas 30-day follow-up visits with the comfort coach are now conducted remotely. We have completed our qualitative interview process and are currently evaluating our coded data, with plans to publish our qualitative findings by the end of 2020. We anticipate that enrollment, data collection, and analysis will be completed by September 2020 and expect to submit our initial quantitative results for publication by the end of 2020.

## Discussion

### Significance and Impact

#### Aim 1: Assess the Effect of a Comfort Coach on Patient Experience

Nonpharmacological interventions administered by a comfort coach have the potential to decrease anxiety, self-reported pain, and stress while improving functional status and overall patient experience in the hospital for cardiac surgery patients, which would mirror findings in pediatric and nonsurgical adult populations [15,19]. These findings would have a significant impact on adult cardiac surgical care and establish a comfort coach role in a multidisciplinary perioperative care team. In addition, these findings justify the dissemination of these techniques and the role of the comfort coach in other types of surgery. Although cardiac surgery elicits significant preoperative anxiety and postoperative pain, other types of surgery such as oncologic, obstetrics, and orthopedic surgery are all associated with high amounts of anxiety, pain, and opioid use [9,40].

#### Aim 2: Measure Differences in Inpatient and Outpatient Opioid Use and Postsurgical Health Care Utilization

The role of surgery in the opioid epidemic has been well described [73] through widespread overprescribing [38,46], with the amount prescribed shown to be the most significant predictor of opioid consumption [47] and development of new persistent opioid use among previously opioid-naive surgical patients [74-81]. In cardiac surgery, persistent opioid use has been shown to confer higher rates of complications, length of stay, and health care costs [37]. Efforts have focused primarily on decreasing opioid prescription [72,82-85], whereas nonpharmacological interventions have not been well described in surgical patients. Comfort coaches may provide a nonpharmacological method for further decreasing opioid use, which would complement and enhance ongoing efforts to decrease prescribing. By addressing concurrent anxiety and decreasing perceived pain, nonpharmacological techniques may serve as valuable tools in addressing the opioid epidemic and improving surgical care.

The effect of the comfort coach’s interventions on anxiety, pain, stress, and opioid use can also be measured through overall health care utilization. As patient anxiety and pain decrease while satisfaction and comfort increase, we expect them to be better equipped and more prepared to leave the hospital. In addition, whereas individual nonpharmacological techniques such as preoperative educational prompts [48,49], massage therapy [50], and music [34] have been tested within hospital settings, no trial has measured the effect of an individualized comfort coach utilizing individualized nonpharmacological techniques throughout the entire perioperative course, from preoperative clinic visit through 90-days of postoperative follow-up. An innovative aspect of this trial is sending different nonpharmacological tools home with patients based on their individualized preferences and continuing self-administered nonpharmacological techniques for 30 days postoperatively. We expect these sustained efforts to reflect decreased health care utilization through hospital length of stay, minimized or eliminated days spent in an extended care facility, prevented emergency room, urgent care, and unplanned doctor office visits, telephone calls, and readmissions, all of which may decrease health care costs.

#### Aim 3: Qualitatively Evaluate the Effectiveness of the Comfort Coach Intervention

We expect the comfort coach intervention to be extremely impactful. In contrast to testing 1 individual technique [50,86-88], the comfort coach intervention is a series of individualized nonpharmacological interventions administered by a trained coach incorporating patient preference and choice from a comfort menu. Qualitative analysis is essential to identify specific aspects of the comfort coach intervention, which were effective or ineffective, and answer *How?* and *Why?* to inform broader implementation. The findings from our qualitative study will prescribe how hospitals can expect to implement our findings efficiently. If the most impactful aspect of the intervention is the individual person, hospitals can focus on providing a companion for patients at critical moments in the perioperative process. If being coached were most impactful for patients, this would justify a certified comfort coach’s role with training in administering these specific nonpharmacological techniques. If patients find specific techniques most effective, these can be packaged into a scalable paper or electronic tool, which can be broadly implemented for surgical patients. Insight into why different aspects of the intervention were effective will guide further implementation.

#### Economic Implications on the Cost of Health Care

Previous evaluations within pediatric [52,53] and adult nonsurgical populations [17,18] have established decreased health care utilization and, consequently, health care costs associated with nonpharmacological interventions. Much of the effect of coaching by certified child life specialists on reducing health care costs has been attributed to the reduced need for anesthesia in imaging procedures [52,53]. Furthermore, coaching interventions have been associated with decreased opioid use duration and length of hospital stay [15-18,51].

We anticipate that our coaching intervention may have more profound impact when evaluated within an adult inpatient surgical population. Relative to nonsurgical and many other types of surgical populations, our cardiac surgical population has greater anxiety, more pain and opioid use, and longer average lengths of stay. Even with our conservative effect sizes estimating a 10% reduction in the published mean episode payments for CABG and valve surgery [30,31], we anticipate savings within Michigan to be approximately US $54 to 80.4 million annually if our comfort coach intervention were disseminated across all 33 nonfederal cardiac surgical hospitals.

By decreasing health care utilization, the comfort coach intervention may have profound economic implications, particularly in decreasing the amount of time patients spend in the hospital. For payers, less health care utilization will decrease episode payments, which reach up to US $15 billion annually in the cardiac surgery population [29-32]. For hospitals, decreasing individual patient health care utilization could mean an increase in new patient admissions and increased efficiency of patient throughput, which carries increased importance in the current era of value-based reimbursement.

### Barriers to the Project

#### Healthy Volunteer and Placebo Effect

Whereas patients receiving the control treatment may report improvement because of the healthy volunteer effect [89], patients receiving intervention may report improvement in anxiety, pain, and satisfaction by virtue of the placebo effect of having a comfort coach. However, we feel that this *placebo effect* is a real effect that we hope to measure through this intervention. Our project’s qualitative component aims to assess what works, what does not work, and even furthermore, how the intervention does or does not work. Accordingly, we feel the potential effect of having a designated coach to be an important measure.

#### Spillover Effects

Our trial may be susceptible to spillover effects, both internally in our intervention and from external forces such as competing institutional interventions. Specifically, opioid reduction efforts for specific general surgery procedures at our institution have demonstrated spillover effects into additional general surgery procedures, with corresponding reductions in opioid use found [83]. Similar efforts around perioperative education and opioid prescribing have taken place in cardiac surgery and may affect the opioid use secondary outcome if current prescribing and use are too low to observe significant changes. Internal spillover effects may also occur, such as nursing and other care team members observing comfort coach interventions and incorporating these nonpharmacological techniques into their usual care. We attempted to mitigate this effect by conducting in-service educational sessions with physicians, advanced practice team members, and nurses to make each stakeholder aware of the trial and the intervention being tested. In addition, if a trend of improvement in the control arm of this trial over time is observed, this difference over time will be treated as the effect size of spillover, and we will report it as such in the trial analysis.

#### Generalizability

If the intervention tested is beneficial, a generalizable implementation of trained comfort coaches for surgical patients may be questioned. To overcome this generalizability barrier, we will first consider our qualitative third aim to describe how these techniques were effective or ineffective. If specific techniques are effective, they can be translated into a scalable paper or electronic tool that can be broadly implemented. Second, the health care utilization is found to be lower among those who receive a comfort coach, we plan to financially quantify this difference with 90-day episode payments between arms and perform a cost-effectiveness analysis incorporating the cost of comfort coaching to demonstrate cost savings that can be used by other health systems to assess whether to implement these methods.

#### COVID-19 Effect

Approximately 20% of patients during the trial will have received inpatient care either during or after the peak of the COVID-19 pandemic. The usual amount of fear and anxiety elicited by cardiac surgery and the associated recovery period enhance patients’ typical need for close physical, emotional, and psychological support from their loved ones. The hospital-wide policy prohibiting all visits during COVID-19 may cause an increase in the levels of fear and anxiety caused by surgery and recovery owing to enhanced feelings of isolation, separation, and possibly even abandonment—at such a crucial time of need—by the most important loved ones in their lives. These policy changes may enhance the impact of a comfort coach on the intervention arm, simply by adding more human contact through a caring and empathetic individual at these critical touchpoints of care, irrespective of the nonpharmacologic therapy the coach provided.

In contrast, the control patient group during the COVID-19 pandemic may be at a potential disadvantage compared with the control patient group before COVID-19 because the pre-COVID control group had the usual level of support from family, friends, and significant others. These 2 opposing effects, namely, a potential enhancement for the intervention group and additional tension and anxiety from isolation and separation among the control group, may have affected the study results. However, it is also possible that patients in the intervention group during COVID-19 remained anxious and fearful despite having a comfort coach because of separation from loved ones, fear of infection in the hospital, or other factors. Although we cannot mitigate the impact of COVID-19, we will perform a subset analysis of patients who received care during COVID-19 to evaluate for any significant differences in our data.

### Conclusions

This clinical trial aims to evaluate the impact of a comfort coach administering nonpharmacological interventions on patient experience, opioid use, and health care utilization compared with usual care in adult cardiac surgery patients. Findings from this study may serve as the foundation for a subsequent multicenter trial, establishment of this role in the adult setting, and broader dissemination of these techniques to other types of surgery.

## Appendix

Multimedia Appendix 1 Supplemental material.

Multimedia Appendix 2 Peer Review Comments and Author Responses.

## Abbreviations

CABG: coronary artery bypass
FCVC: Frankel Cardiovascular Center
GAD-7: Generalized Anxiety Disorder 7-item scale
IES-R: Impact of Events Scale-Revised
KCCQ-12: Kansas City Cardiomyopathy Questionnaire short-form
OME: oral morphine equivalents
PHQ-9: Patient Health Questionnaire-9
TATUM: Treatment Assignment Tool-University of Michigan
