# Supplementary material for: The Impact of Nonpharmacological Interventions on Patient Experience, Opioid Use, and Health Care Utilization in Adult Cardiac Surgery Patients: Protocol for a Mixed Methods Study
Source: JMIR Res Protoc. 2021 Feb 16;10(2):e21350. doi: 10.2196/21350 (PMC7925147; doi:10.2196/21350)

**SUPPLEMENTAL APPENDIX**

**Comfort menu.**

**
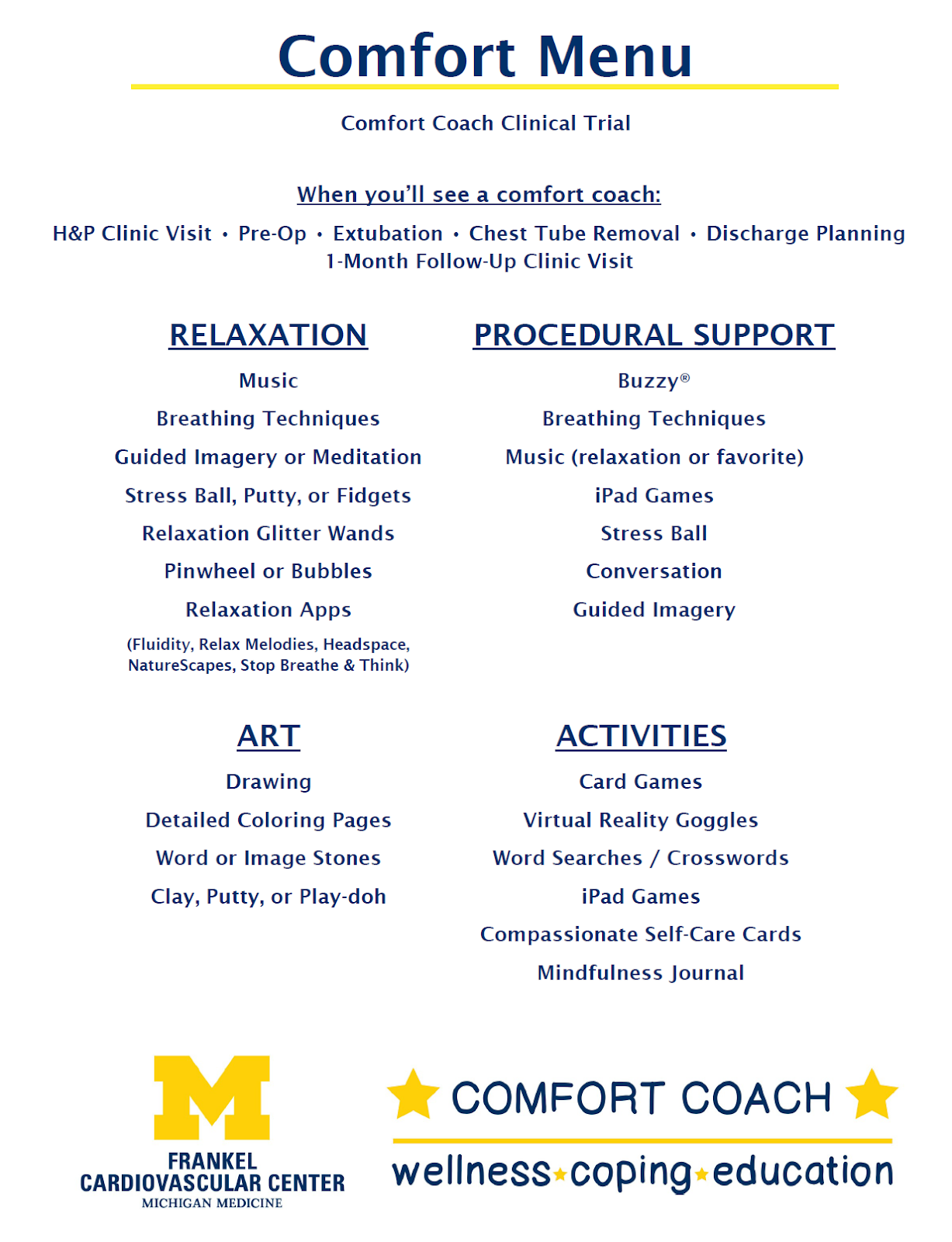
**

**Generalized Anxiety Disorder 7-item Scale.** A 7-item validated questionnaire to assess and potentially diagnose generalized anxiety disorder [56].

**
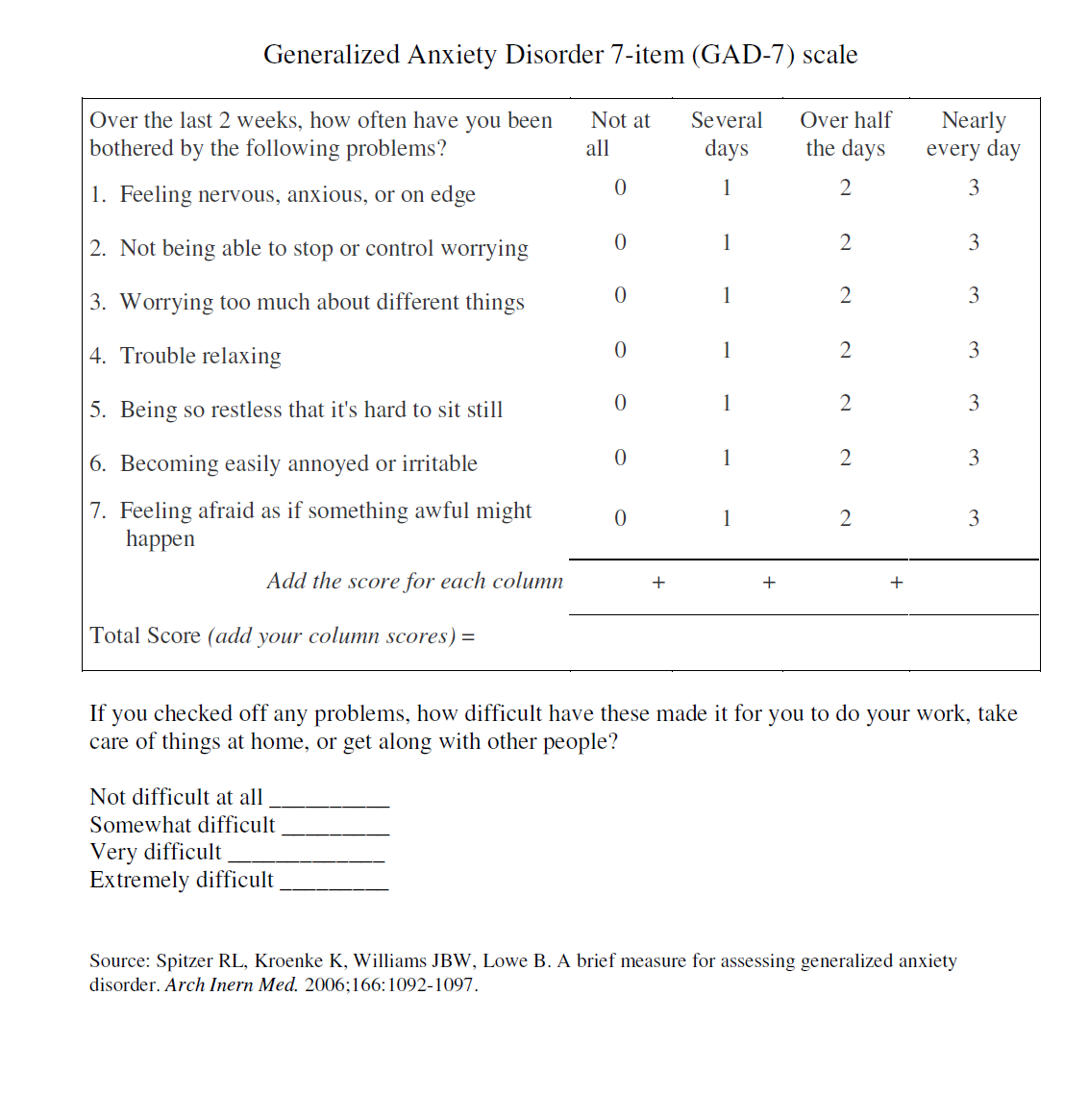
**

**Patient Health Questionnaire.** A 9-item validated questionnaire which generates a total score out of 27 used to diagnose 5 different degrees of depressive disorders [57].

**
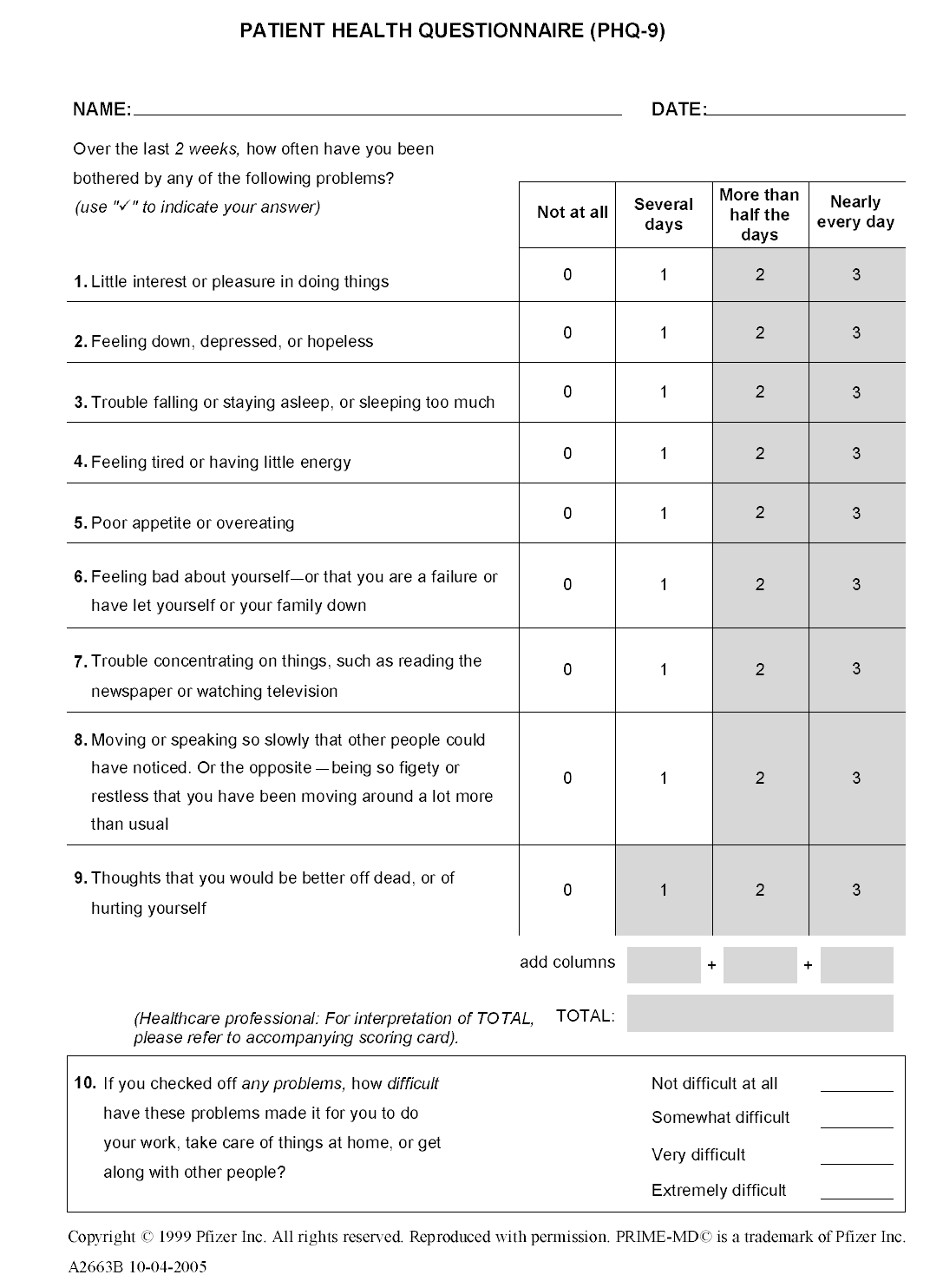
**

**
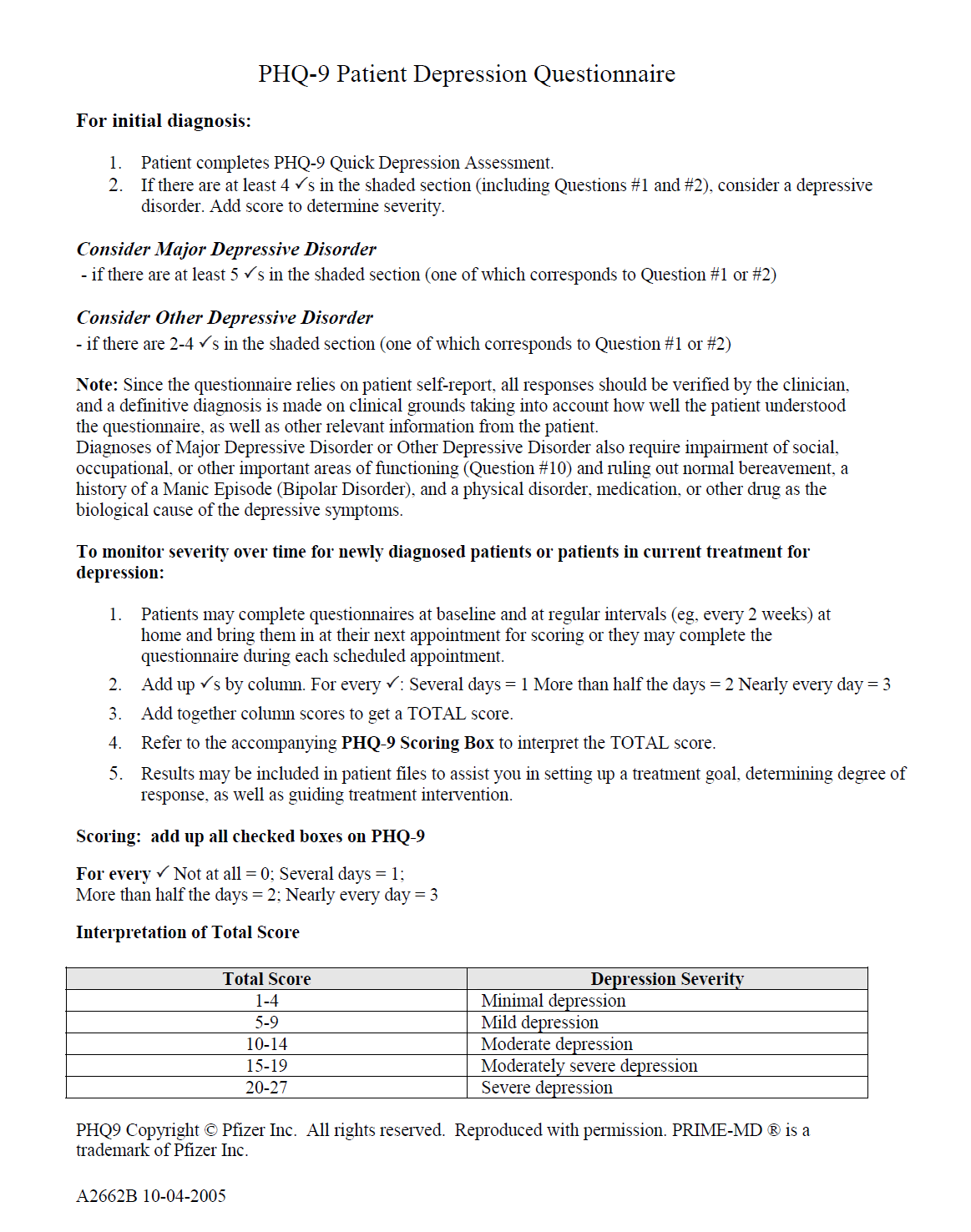
**

**Impact of Events Scale-Revised.** A 22-item validated scale utilized to measure event-related stress with the potential to indicate clinical suspicion or diagnosis of post-traumatic stress disorder [60].

**
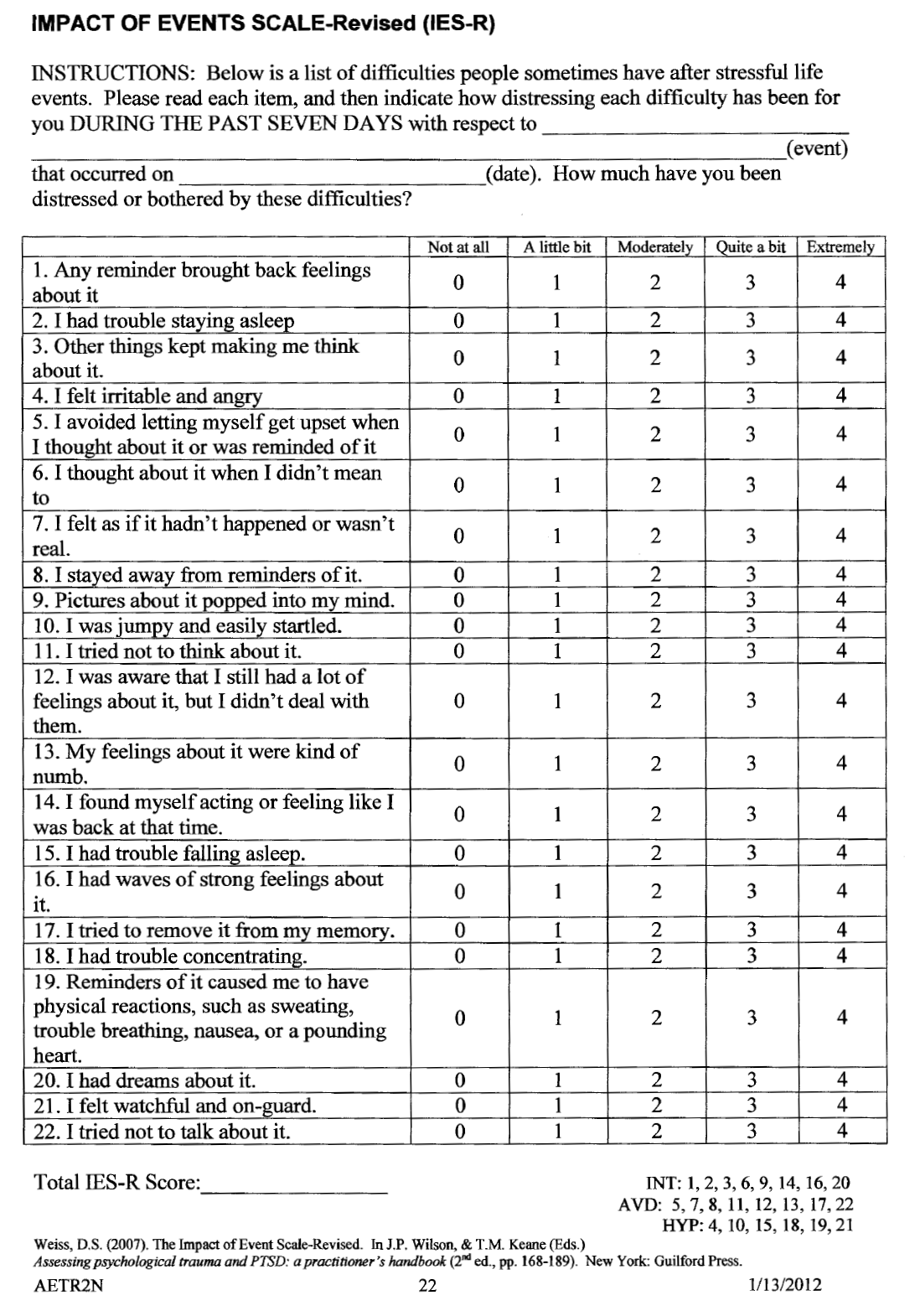
**

**
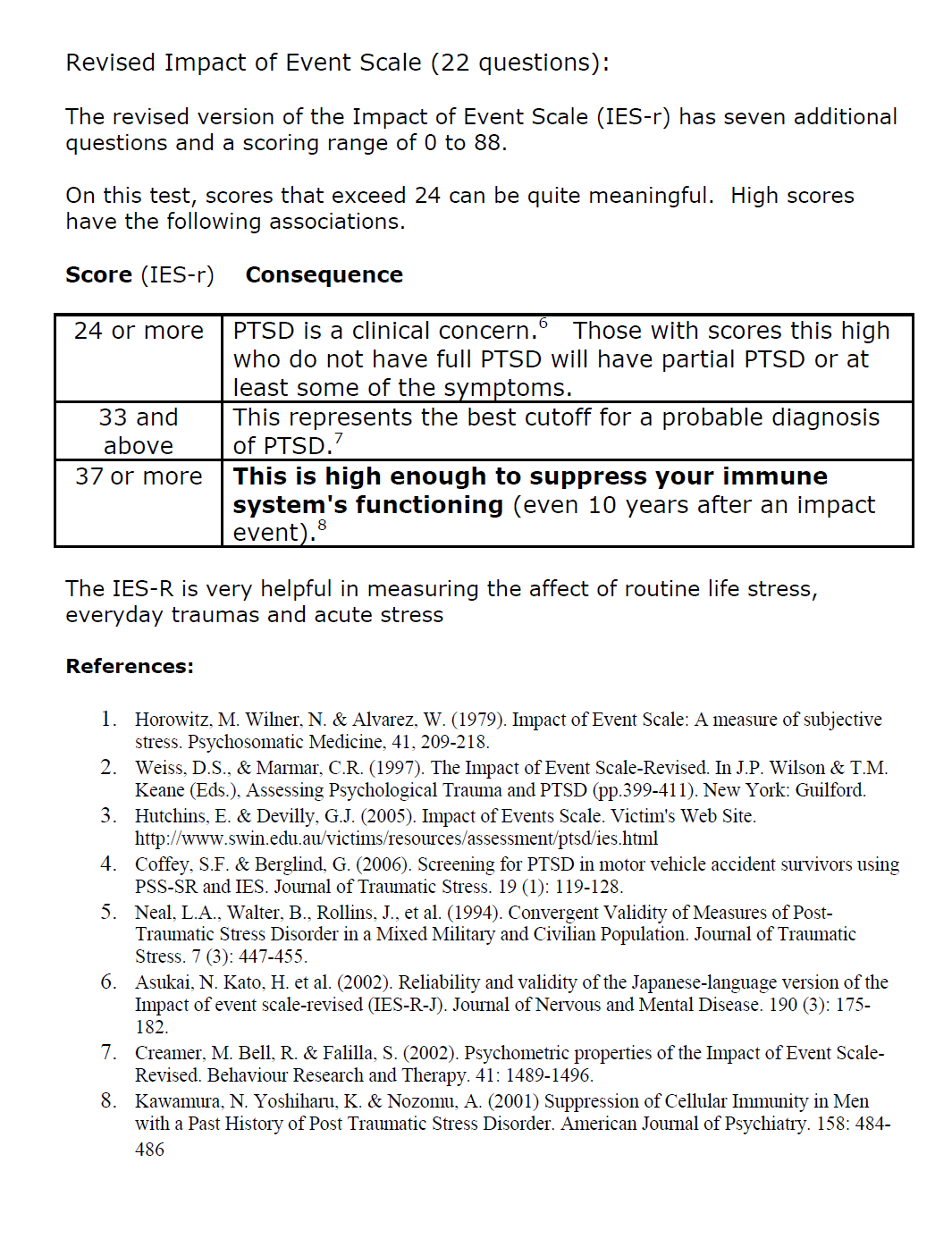
**

**Picker Patient Experience Questionnaire.** A 15-item questionnaire designed to capture the patient’s inpatient experience [61].

**
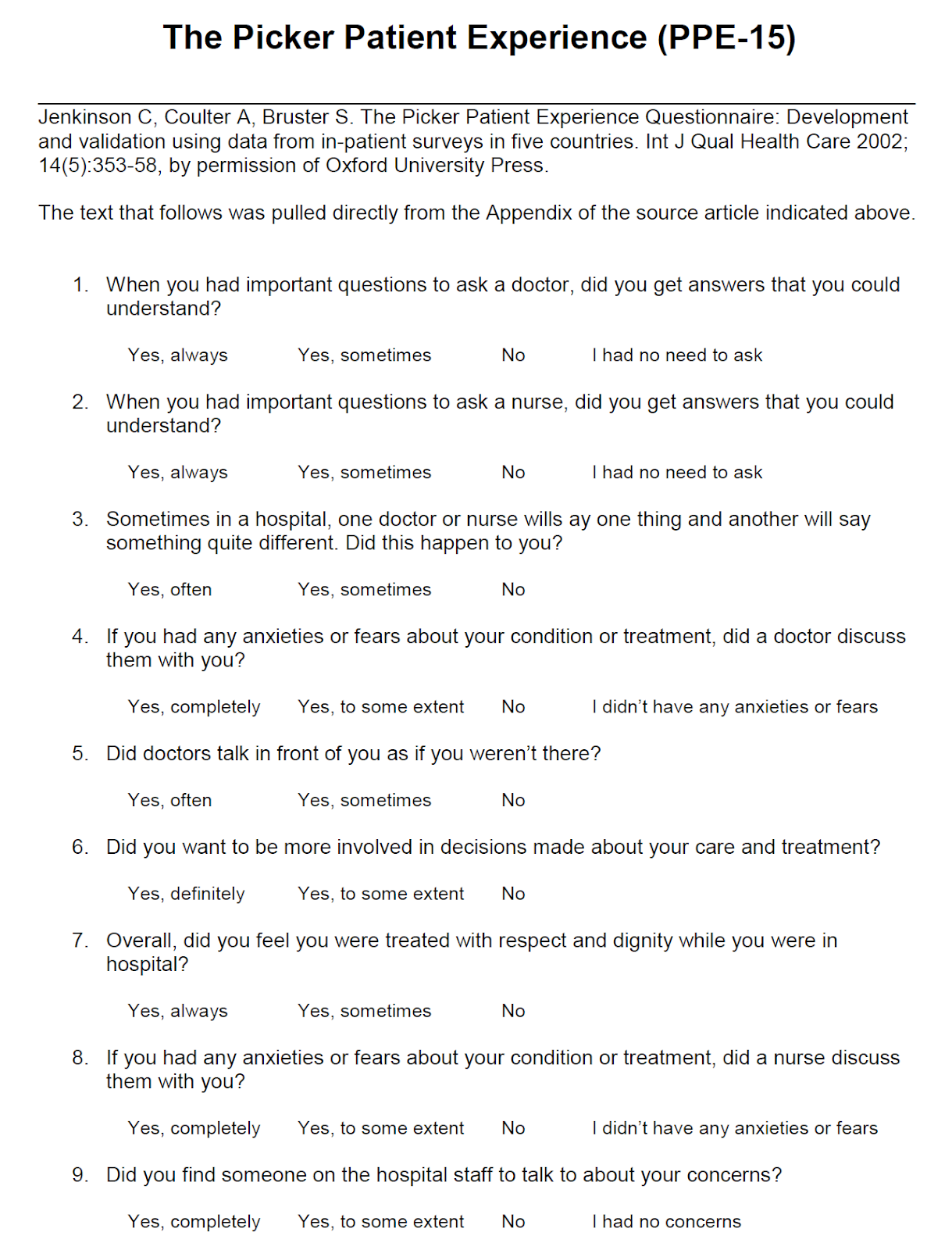
**

**
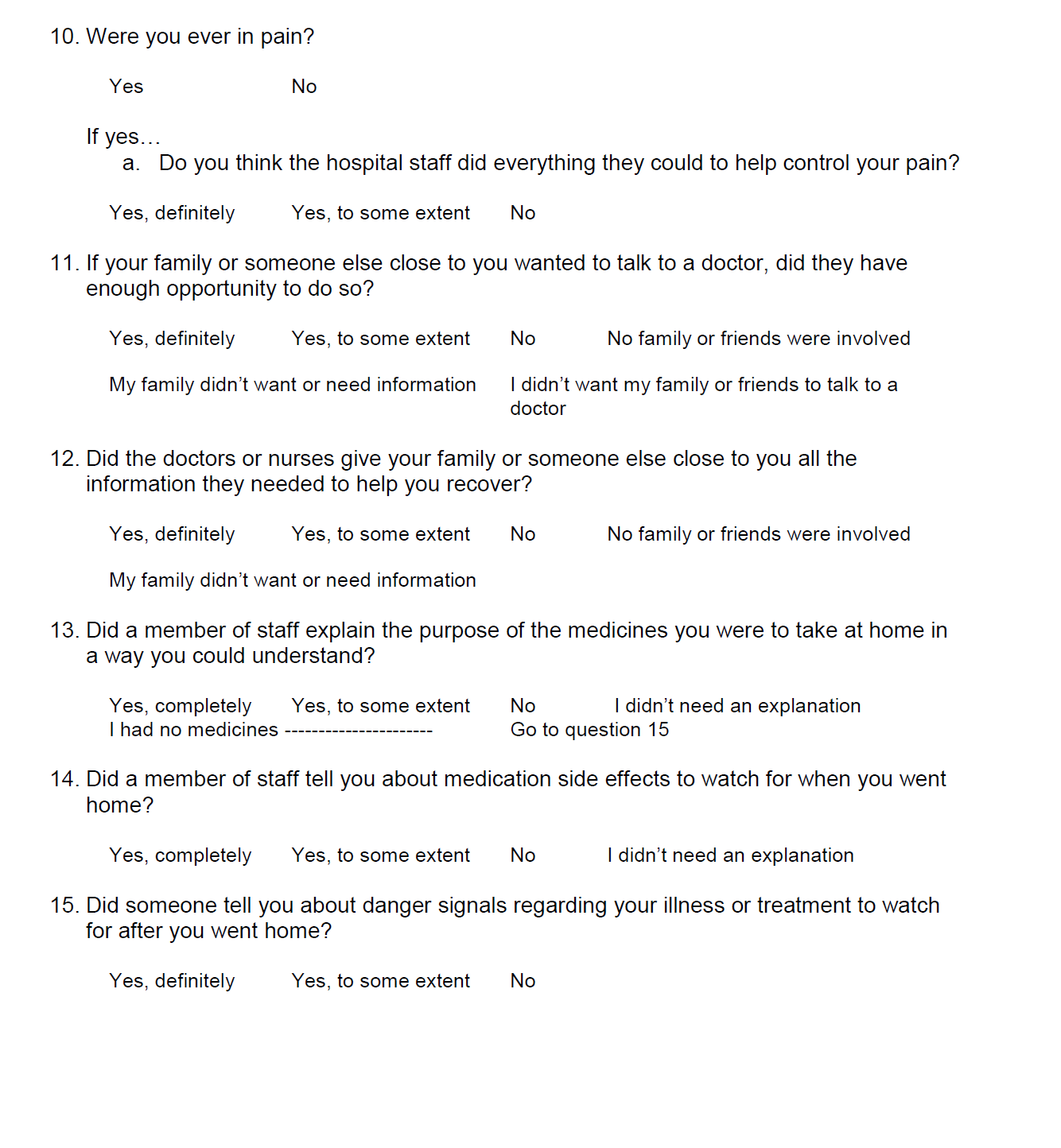
**

**Brief Pain Inventory.** Short-form 9-question inventory assessing patient pain location, severity, relief, and activity level [62-63].

**
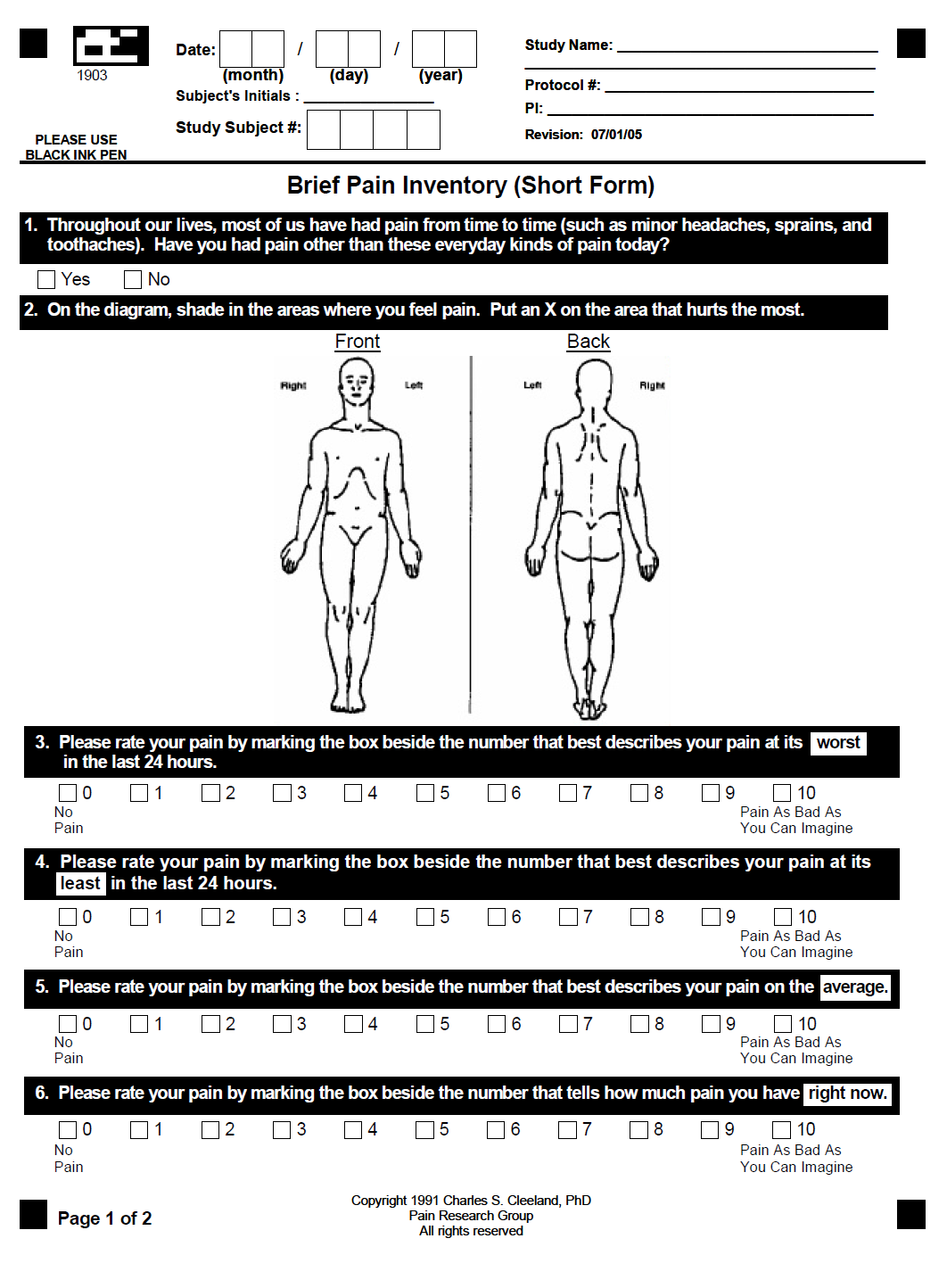
**

**
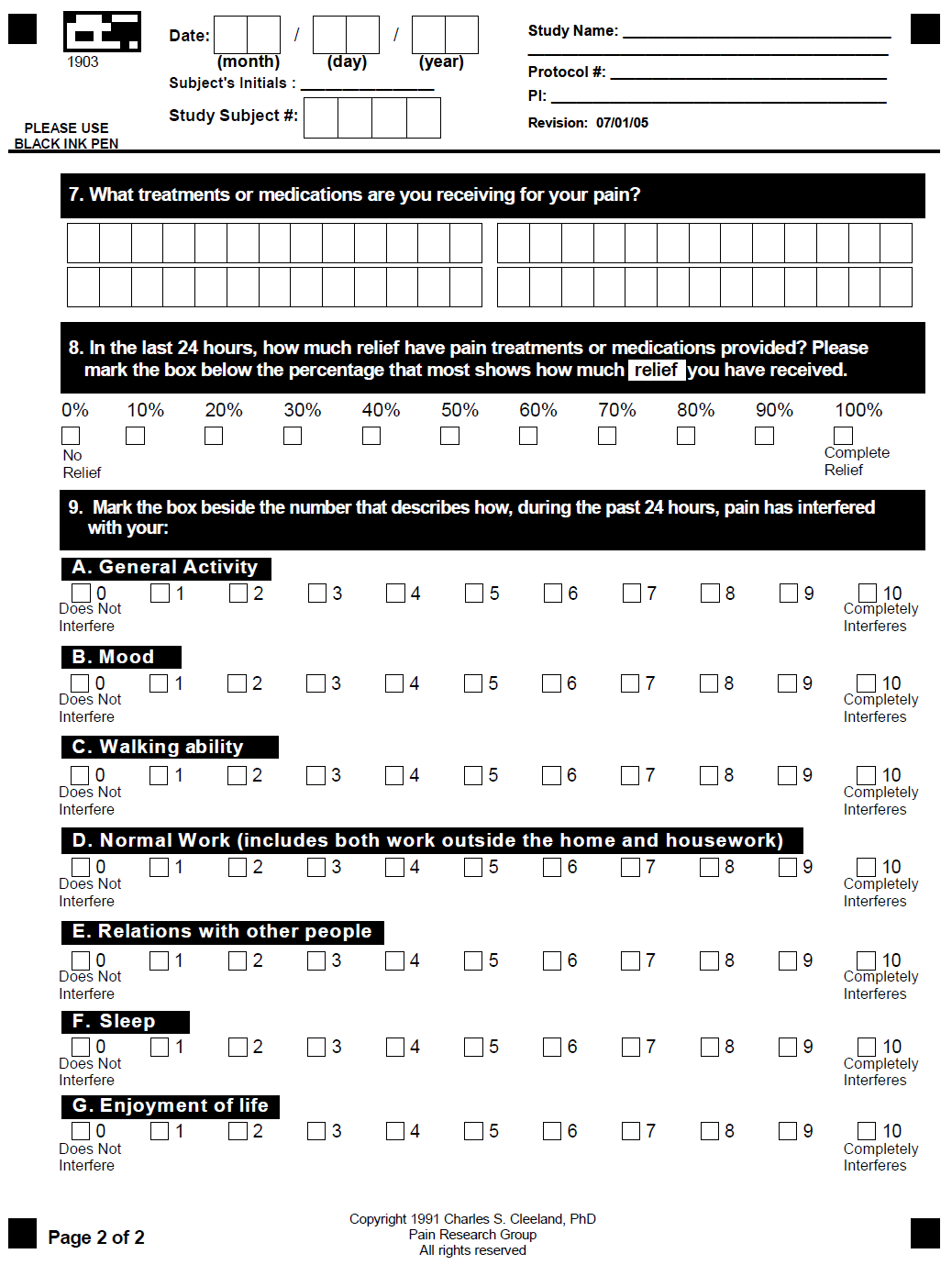
**

**Postoperative Opioid and Pain Management Questionnaire.** 11-item questionnaire collecting data on opioids prescribed, opioids used, pain scores, opioid storage and disposal practices, and assessment of opioid education.

**
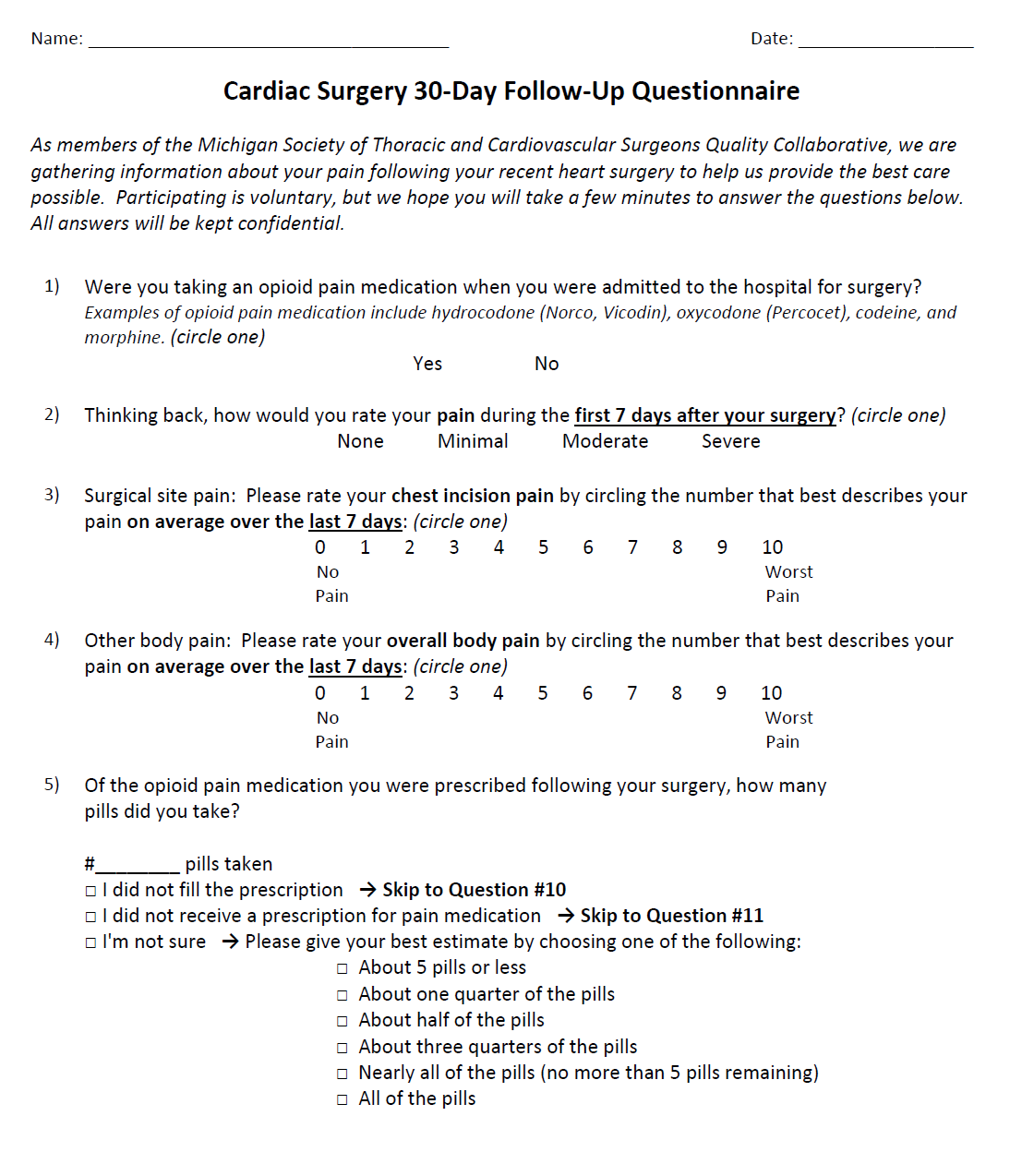
**

**
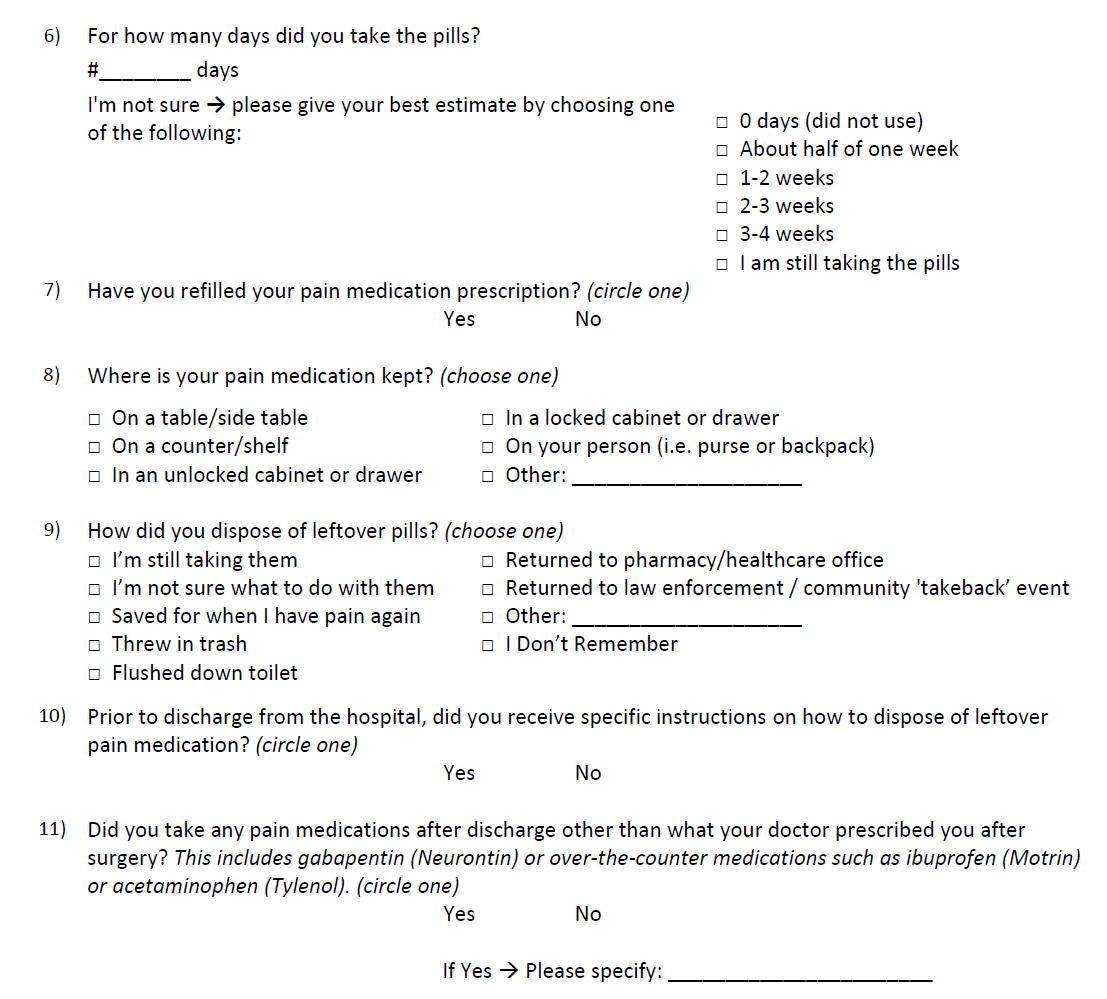
**

**Kansas City Cardiomyopathy Questionnaire.** 12-item questionnaire assessing the impact of heart failure on the daily activities and lifestyle of the patient [58-59].


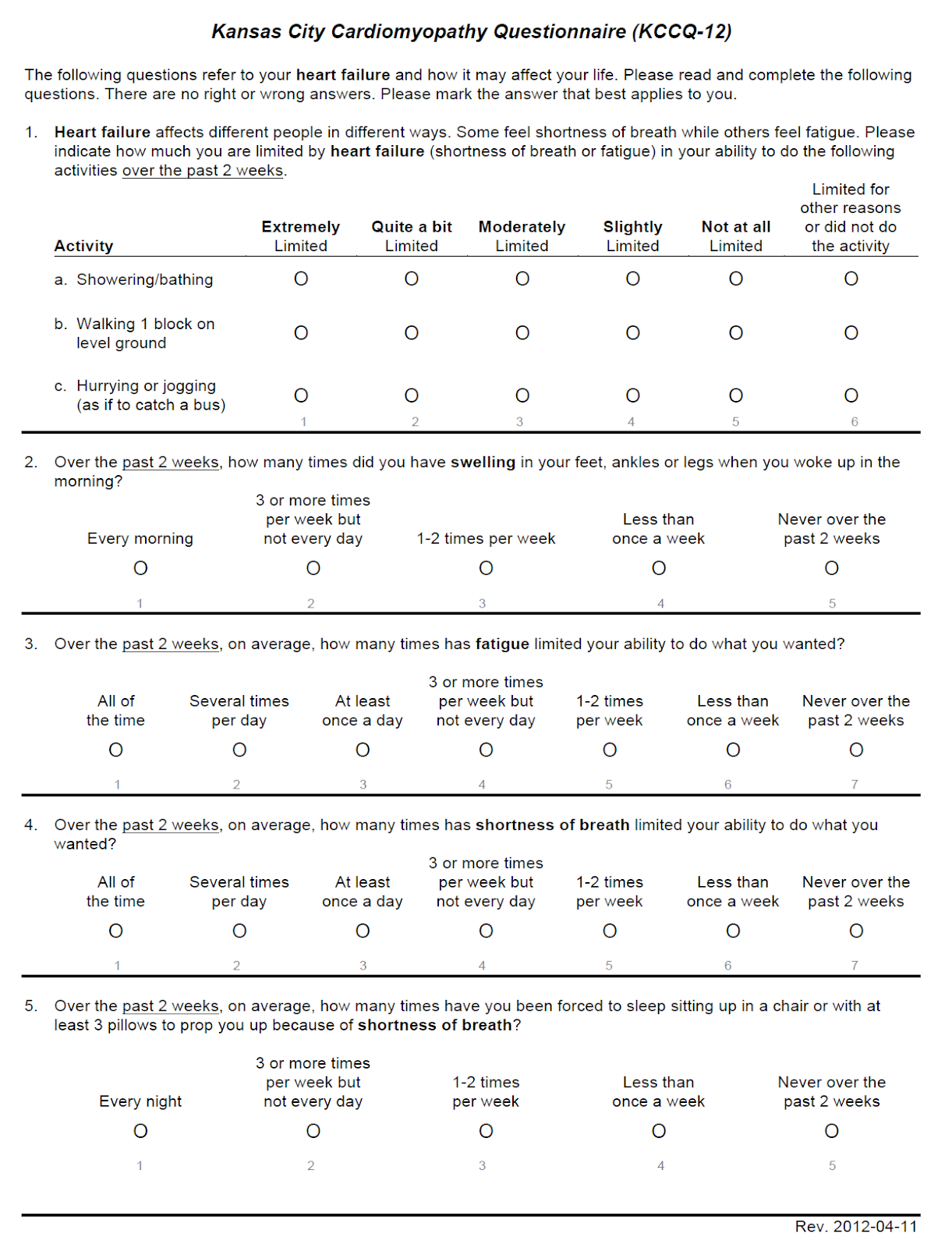


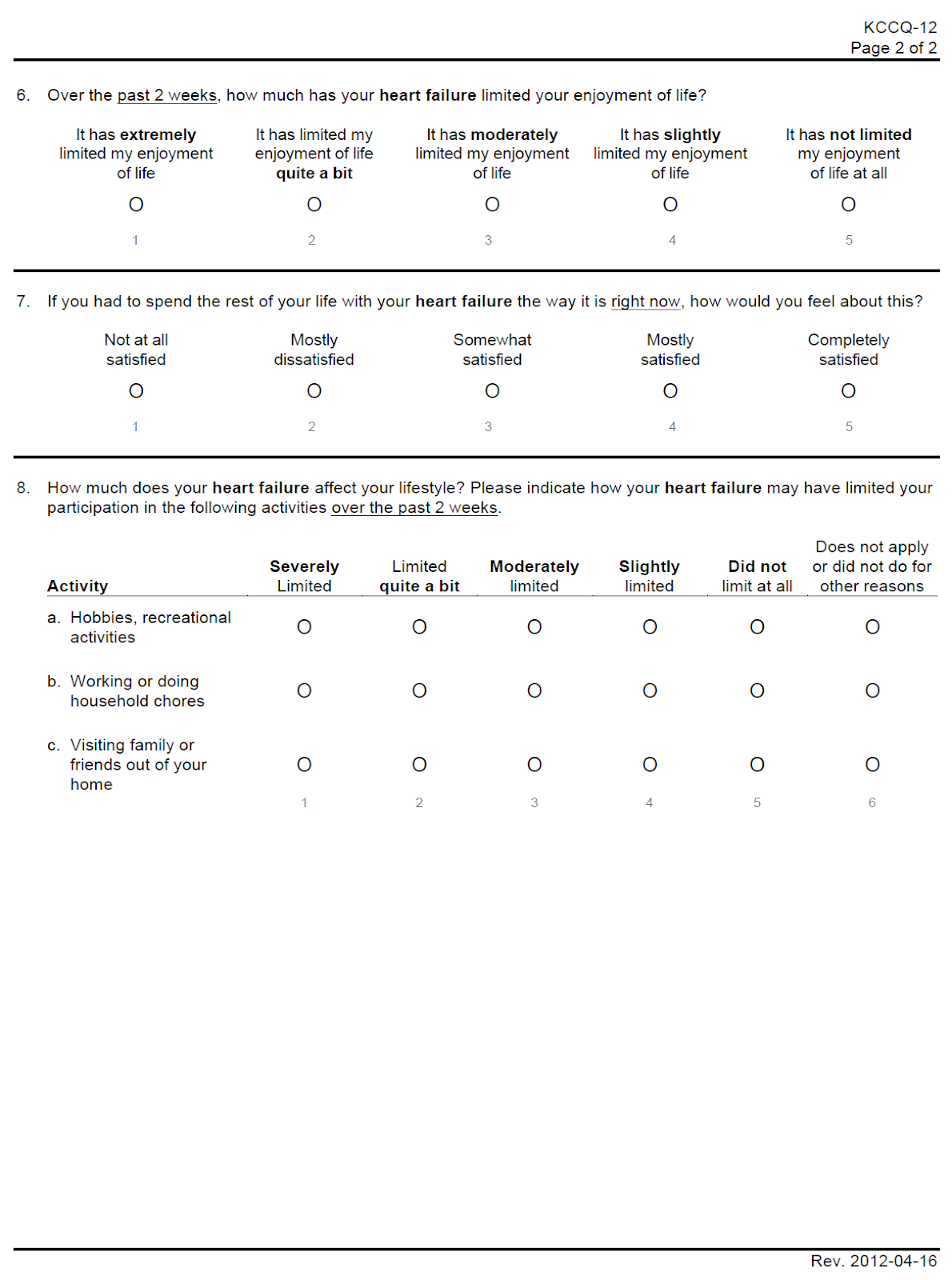

Supplement: Multimedia Appendix 1 [file resprot_v10i2e21350_app1.docx]
