# Supplementary material for: The Impact of Nonpharmacological Interventions on Patient Experience, Opioid Use, and Health Care Utilization in Adult Cardiac Surgery Patients: Protocol for a Mixed Methods Study
Source: JMIR Res Protoc. 2021 Feb 16;10(2):e21350. doi: 10.2196/21350 (PMC7925147; doi:10.2196/21350)
Supplement: Multimedia Appendix 2 [file resprot_v10i2e21350_app2.pdf]

BCBSM Foundation
Reviewer Comments: 2812.ii

Proposal # 2811: **The impact of non-pharmacological interventions on patient experience, opioid use, and healthcare utilizations in adult cardiac surgery patients**

Reviewer #1

Description:

The PI plans to test whether the addition of a comfort coach who will work through a self-selected set of psychological exercises with each patient will improve patient experience and use of prescribed opioid medications for pain management and health care utilization during the first 30 days post-cardiac surgery.

Review:

Comment #1: The tracking of pharmaceutical pain management seems to be limited to opioid use and, as such, will be converted to oral morphine equivalents. It is not clear why the investigators will not be tracking other prescription pain management drugs, or non-prescription means that the patient may employ.

> **Response #1**:
> This is an excellent point. While we have focused in our Aims and analysis on both inpatient and outpatient opioid use, **we will also capture non-opioid adjunct pain medications on our 30-day postoperative questionnaire, included in the Appendix.** This form includes a question about adjunct pain medication use (yes/no), as well as type, including prescribed gabapentin, as well as over-the-counter acetaminophen and ibuprofen. **In addition, we agree that all pain management drugs should be captured and therefore we will also record whether adjunct medications were utilized by subject as an inpatient, the type of medication, and the amount taken.**
>
> **In addition to pharmacological pain management strategies, we will be tracking the 6 targeted interventions by the comfort coach for patients in the intervention arm, including comfort coaches listing the specific non-pharmacological techniques utilized by each patient at each time point.** These data will be recorded in the comfort coach's standard visit note and utilized to inform and guide individualized care. In addition, during 1-month and 3-month follow-up we will plan to inquire and record whether or not patients are utilizing the same non-pharmacological strategies at home that they were administered by comfort coaches while hospitalized.
>
> **In aggregate, these data points will provide the complete picture of both pharmacological and non-pharmacological strategies for pain management throughout the postoperative period.**

Comment #2: It is unclear that there is a standard protocol for prescribing pain meds post-op. Will both groups have the same opioid and non-opioid prescription regimen? Do all patients receive the same opioid education and is this a current standard?

> **Response #2**:
> **With individualized exceptions, patients undergoing cardiac surgery through a sternotomy at our center typically receive 28 pills of 5 mg oxycodone to take every 6 hours as needed in conjunction with scheduled acetaminophen 1000 mg every 8 hours.** Patients are instructed to use the least amount of narcotics necessary for breakthrough pain not controlled by Tylenol. We emphasize they can wean narcotics to take the least amount necessary to have tolerable pain to

the extent they are still able to walk and deep-breathe sufficiently. Furthermore, they are told not to keep left-over opioids, but to take them to a designated take-back facility.

In addition, patients receive a standard preoperative manual detailing each step in the cardiac surgery experience, as well as targeted opioid-related preoperative education. Preoperative education includes a standard script of talking points reviewed with each patient which is based on the “start talking” Michigan state consent form for opioid prescribing. In addition, education regarding new Michigan opioid prescribing laws is provided and patients are told that most patients are able to wean off narcotics and onto non-opioid alternatives within approximately one week of discharge. Patients are also instructed that if they need a refill of opioid medication after one week, they are required to return to clinic and be seen in person, to ensure they do not have a wound or sternal bone dehiscence which could be causing persistent pain. Finally, we review with patients that some postoperative pain is from musculoskeletal guarding and therefore reinforce the need to do postoperative stretching exercises, which are outlined in the book they receive.

This regimen is the current standard for all patients, administered entirely by existing cardiac surgery clinic personnel, and will not be altered by the investigators of this study in any way. These existing standard protocols will be utilized for both intervention and control arm patients.

Comment #3: It is unclear what number of adult cardiac surgery patients (if any) will have experienced childhood surgery p. 13-14). Therefore, it is unclear what relevance the PI’s discussion of such experiences has to the proposed project. Why wouldn’t an adult comfort coach be a more appropriate intervener, or is one not available to the research team?

Response #3:
This is an excellent point raised by the Reviewer. Our intent in citing childhood surgery was to describe an example of one mechanism of surgery-related stress to illustrate the skillset of the child life specialists, rather than to assert that the patients in this trial will have had surgery in childhood. The cited discussion presents existing evidence in the literature about the adverse effects of unresolved trauma resulting from surgery which can recur and be provoked by stressors such as cardiac surgery. **This specific point is part of our larger contention that these specialists are ideally trained for this role in providing comfort to our adult cardiac surgical patients.**

Importantly, the role of a trained, dedicated, adult comfort coach does not currently exist in any healthcare system to the knowledge of our team. However, non-pharmacological techniques administered by these specialists to reduce pain, anxiety, and stress have been shown translatable to the adult population, but have not been assessed in an adult surgical population. This gap in practice motivates the proposed trial, in hopes of improving cardiac surgical care as detailed in our proposal.

Comment #4: The four-point pain scale (Question 2 on the one-month post-op questionnaire) is an ordinal variable and should be analyzed as such. Using a chi-square for this scale really isn’t appropriate and information may be lost if analyzed this way.

Response #4:

Thank you for bringing this to our attention. For the four-point pain scale, we will use Mann-Whitney U tests to compare the two groups of ordinal data.

Comment #5: The PI should be aware that standard deviation is a descriptor of normally distributed distributions and that the t-test can produce very misleading results when used on seriously non-normal data. In the power analysis discussion (p. 18), with targets of mean reduced opioid use and standard deviations, this is obviously not the case for the cited data. To encompass 95% of a normally distributed sample ($\pm$2 standard deviations) with as mean of 140 OMEs and standard deviations of 125 OME or 100

OME, would require that many folks would be significantly increasing opioid use: 140-250 = -190 OME. Unless the PI is anticipating increased opioid use. Therefore, anticipating such an obviously skewed sample, it would benefit the PI to consult with a statistician for the appropriate method of non-parametric statistical analysis.

Response #5:
Thank you for this thorough assessment. We initially intended to use parametric statistics since our data will likely satisfy the central limit theorem; however, prior literature, statistician consultation, and the Reviewer's comments are well-taken and we will plan to utilize the appropriate non-parametric statistical analysis instead.

In this instance, we will quantify amount of opioid prescribed and used in median and interquartile range of OME and will use the Mann-Whitney U test to compare quantities. Subsequent to this grant submission, the described statistical methods have been confirmed by our statistician consultants and published by our group for similar data (Howard et al. JAMA Surgery 2019;154(1):e184234).

Comment #6: In tracking the number of days spent at home, a crude proxy for health care utilization, it is possible that subjects will be out-of-home during the period near and through the end of that 30-day window. I'm not sure how the PI might best handle this possibility, but I just note it.

Response #6:
While we have defined this composite endpoint as days at home within the first 30, we are interested in capturing healthcare utilization during the initial postoperative period. **We define "home" as returning to a place of residence which does not require additional healthcare resources and spending for the patient to reside there. If patients reside in an assisted living community, then returning to this community post-discharge would be considered days at home.** We do not anticipate patients entering the hospital or a different facility for medical purposes, both unrelated to their cardiac surgery and within 30 days after surgery, but it is possible this could occur.

Comment #7: Will all subjects be covered through BCBSM, Medicare or Medicaid (use of Michigan Value Collaborative Data) (p. 17).? If not, how will subjects covered through other plans be handled?

Response #7:
This is an excellent point. Our primary outcome in this trial will be postoperative healthcare utilization as defined by our composite outcome, with secondary outcomes of inpatient and outpatient opioid use, patient experience, anxiety, depression, and stress. If we find a difference in postoperative healthcare utilization between groups, we will only then plan to quantify these differences by comparing total and component episode payments between groups. We plan to perform this economic analysis in the subset of patients with data available through the Michigan Value Collaborative, which includes Medicare fee-for-service, BCBSM PPO and Medicare Advantage, Blue Care Network HMO and Medicare Advantage, or Medicaid, which we anticipate to be at least 80-85% of patients. **We feel that these payment data, even as a majority subset, will be superior to assessing hospital charges. Additionally, excluding patients without these specific payers would bias the main outcomes of the trial and would not be justified. While the analysis will likely not capture 100% of patients, we can evaluate any bias that exists and this specific analysis will represent the largest analysis of this type available to date.**

Personnel:

Comment #8: Named research personnel are highly qualified, although the role of many of the named personnel are as research mentors to the PI, and no as individuals with specific project assigned duties.

No funding is being requested for the actual researchers. Funding requests are for the person actively providing the intervention and temporary research assistants.

One piece of information that has not been included is the amount of effort each of the named personnel will be committing to this project. Without such information it is impossible to judge whether their efforts will be appropriate and sufficient to carry-out and complete the proposed work.

<u>**Response #8**</u>:
Note: As I mentioned on our call, some of the language below was provided by Marlie Bartow, our pre-award officer from the Surgery Research Office, and Heather Offhaus, the Director of Grants from the UM medical school. As I also reiterated, **please let me know if you need a letter of support from any of the mentors or consultants listed on our budget to indicate their level of support and commitment for this study.**

This is an excellent point and I am happy to provide more detail about personnel commitment to this project.

As PI, my individual effort and time will be provided through my T32 training grant. This grant, funded by the NIH, provides 100% protected time for research. The BCBS project will be the main focus of the upcoming year of training time and will include my effort in providing the necessary direction, team support, research design, and commitment to successfully complete the project. I am also supported by personnel and resources by virtue of my position as a research fellow at the Center for Healthcare Outcomes and Policy (CHOP) and the Institute for Healthcare Policy and Innovation (IHPI).

Julie Piazza, co-investigator, serves as the Senior Project Manager in the Office of Patient Experience. As a certified child life specialist and the original pioneer of this trial from the inception of the feasibility trial, Julie is heavily invested in supporting me and the entire group in this endeavor. We have a commitment that her role in the Office of Patient Experience will benefit the program outlined in the proposal, but we do not currently have a way to quantify the exact amount of time that will be spent specifically within this statement of work. However, without Julie, this project would not exist and her continued close involvement is essential to our success. My level of contact with Julie specifically for this project includes in-person meetings 1-2 times per week and daily communication.

The University of Michigan Medical School recognizes mentoring effort of faculty up to 5% of their time concurrent with their ongoing research activities. Each mentor will serve in an advisory capacity as experts in their field, sharing guidance in their area of expertise within their mentoring time.

Dr. Deeb is the lead faculty mentor for this grant and with Julie Piazza has helped lead and guide this project since its inception through our feasibility trial. The effort and financial support committed to this project by Dr. Deeb are substantial. Specifically, the funding of the research coordinator and research assistant will be covered by the Department of Cardiac Surgery through Dr. Deeb's discretionary research budget. My level of contact with Dr. Deeb for this project includes in-person meetings 2 times per week and near-daily communication.

Dr. Likosky is currently my main research mentor on my T32 training grant. Through this fully-supported training grant, Dr. Likosky serves as my mentor in an official capacity for all of my research activities, including but not limited to this project. Accordingly, my level of support from Dr. Likosky includes in-person meetings multiple times per week to discuss research activities, as well as frequent, constant, and dedicated feedback on methodology, content, and writing.

The remaining mentors and consultants are all experts in their fields which are highly relevant to the current project, as detailed in in the Budget Justification. Each have been readily available and involved when needed and have offered substantive support and input into this grant proposal.

Budget and Timeline:

Comment #9: The budget appears appropriate to support the budgeted tasks.

Assuming that subject recruitment will be as successful as hoped, the timeline looks reasonable. However, it has been my experience that "approaching 10-15 patients per week" (p. 13) frequently does not mean "enrolling" 10-15 patients per week., especially when they will be told of randomization and their likelihood of ending up in the no-coach group. And, given their 2017 census data, the investigator will need to successfully enroll from about 33% (approach 15/wk for 7 months) to 70% (approach 10/wk for 5 months) of eligible patients to meet their sample size target. These may be ambitious projections.

Response #9:
We appreciate this point since without a realistic expectation of participant enrollment, this study would face challenging barriers. Although a small sample size, we approached 12 patients for our feasibility study: 11 consented and were enrolled, while 1 was excluded due to requiring a foreign language translator, and 0 patients chose not to enroll due to fear of the "no coach" group or any other reason. While we remain cautious in our estimates, we consider enrolling an average of 6 patients per week for a 6-month period to be extremely conservative, representing our lowest estimated number per week and a modest interval of time.

Recommendation:

The Pi proposes an interesting approach to improve patient outcomes post-cardiac surgery and any linked decrease the amount of opioids needed for short-term (30 day) pain management could have a positive effect on the likelihood of longer-term opioid dependency.

One of the aims of the project, that of examining the effect on healthcare utilization and the financial implications of such a reduction is the weakest part of this proposal.

The PI should be able to easily address most of the above cited points. If done so satisfactorily, support should be considered.

Significance	23
New Information	14
Technical Quality	10
Qualifications	10
Feasibility	10
Total	77

Reviewer #2

Thank you for the opportunity to review Dr. Brescia's proposal entitled -The Impact of Non-Pharmacological Interventions on Patient Experience, Opioid Use and Healthcare Utilization on Adult Cardiac Surgery Patients. This project will explore interventions that have been successful in managing acute and chronic pain in other patient populations to those in the adult cardiac surgery setting. Impacts may include improvement in quality of life, lowered risk of opioid dependence and cost constraint. This is a proposal well aligned with the Triple Aim.

The proposal clearly described significance and the long term goal of using these results as pilot support for a study more broadly generalizable across adults with other surgical conditions. Furthermore, the

authors address sustainability in the plans to address fiscal results that will allow for including the Comfort Coach in the routine care of clients. The methods described for the quantitative segments of the project (Aims 1&2) are clearly described. Material to be used by the "Comfort Coach" are included. as well as description of surveys and chart data to be elicited at each point in the project.

Comment #10: I do have several questions as I review the exclusion criteria that it may help the investigators to consider prior to enrolling subjects. Exclusion criteria includes "medical and psychiatric conditions precluding participation". This is very vague as to which comorbid and concurrent therapeutics other than opioids would cause exclusion. More specific direction would facilitate consenting by the staff.

> **Response #10**:
> Thank you for bringing up this point. Since submission of the grant, we have added Michelle Riba, MD, PhD, and Melissa Webster, LMSW, to our study team. Dr. Riba is a Professor of Psychiatry, while Melissa is the Administrative Manager for Psychiatry at Michigan Medicine's Depression Center**. Following their recommendation approximately two months ago, we have removed these vague exclusion criteria and will not implement them in this trial.**

Comment #11: Will the status of "opioid-naive" be determined by self-report only or confirmed by urine drug screen (which would also have budget and human subject considerations)?

> **Response #11**:
> **The status of "opioid-naïve" will be defined as not taking opioids at the time of preoperative clinic history and physical exam and will be determined by self-report and electronic health records.** We do not feel urine drug screen to be necessary for these purposes and **will adhere to the same definitions and information standards as existing literature on preoperative opioid status and use in surgical patients.**

Comment #12: The criteria for healthcare utilization is post-op days at home. Will adults who live in communal residential settings be included especially those in Assisted Living or SNF prior to surgery.

> **Response #12**:
> Thank you for bringing this question to our attention. **The purpose of our composite endpoint of healthcare utilization is to capture healthcare-specific settings which require healthcare spending, rather than communal residential assisted or independent living settings**. Additionally, the overwhelming majority of patients undergoing cardiac surgery will not be in a skilled nursing facility prior to operation and still be fit to undergo it, especially a sternotomy as is performed for patients in this trial. **Further, patients arriving directly from another healthcare facility will already be excluded, as these will not be elective admissions.**
>
> **For the purposes of this trial, we will only count a post-discharge location with escalated acuity compared to preoperative location as contributing days not at home for our composite endpoint of healthcare utilization.**

Comment #13: The research team is highly experienced and has demonstrated a commitment to support Dr. Brescia's development as a researcher over time. The mentors are well qualified in research related to CV surgery, opioid use and abuse, and non-pharmacological interventions. Co-Investigator Ms. Piazza is well qualified to support the implementation of the "Comfort Coach" role intervention. Dr. Likosky appears to be the mentor providing most support in methods and statistics. While he has extensive experience to support the quantitative portion of the study, his experience in qualitative

methods and design appears more limited. If a rigorous qualitative analysis is planned for Aim 3 perhaps an additional consultant might add strength to the team.

Response #13:
Thank you – we completely agree with this point. **We have formally added Mary Byrnes, PhD, as a Consultant for this project for. Dr. Byrnes is a qualitative methods expert based in the Center for Healthcare Outcomes and Policy (CHOP) within Michigan's Institute for Healthcare Policy and Innovation (IHPI), in which I am currently a research fellow and will have the opportunity and support to work closely with Dr. Byrnes as part of her dedicated, protected time as a consultant for research fellows.**

Comment #14: This is a well-crafted proposal but I have identified two areas of concern that the authors may want to consider prior to beginning the study. The methods planned for Aim3 are much less developed than those for Aims 1&2. The only description of data collection strategy is a "semi-structured interview". No draft of the script is included nor discussion of who will conduct interviews and analyze these data. Again consideration of this component of the project would lead to additional budget for personnel, transcription and qualitative software. If the authors' intent for the analysis of Aim3 is strictly project evaluation of their plan for quality improvement purposes formal qualitative method approaches may not be necessary but if they truly want to conduct "thematic analysis" (page 11/17) then the rigor of the data collection and analysis and related costs must be considered.

Response #14:
This is an insightful point and we are happy to add more detail. We will execute the qualitative portion of this study (Aim #3) under the expert guidance of Dr. Byrnes. **Notably, as a CHOP and IHPI research fellow, I not only have access to Dr. Byrnes protected time as a consultant for this project, but also to a number of important qualitative research tools, including but not limited to: MAXQDA software (qualitative data analysis), NVivo software access, dedicated medical student assistants, and multiple encrypted voice recorders. Our specific plan is to follow a thematic analysis framework as outlined by Braun and Clarke (2012) including semi-structured interviews conducted by 3-4 investigators and led by Dr. Byrnes. We will conduct team coding of transcriptions during which we will each read 3 transcriptions, then meet to discuss the data and identify variables within the answer. From this we will develop a codebook and validate the codebook on the same transcripts, after which we will finalize a codebook. Finally, we will meet and analyze the in vivo codes to draw conclusions. This process describes a validated approach to performed a rich thematic analysis, for which I am ideally positioned to perform with Dr. Byrnes and the resources available through CHOP and IHPI.**

Comment #15: The second issue for consideration is also in the area of budget. All staff expect the Comfort Coach and RA are contributed with no percentage of effort noted in the budget. Neither of the intuitional letters of support explicitly assure the personnel costs are covered and it is unclear if Ms. Piazza's role is in the same administrative chain as Dr. Brescia. The protocol requires 6 face to face interventions between the Comfort Coach and the subjects some of which i.e. time of extubation cannot be planned far in advance nor does it always occur during normal work hours. The proposal calls for approximately 400- 450 interventions. The proposal only asks for 1 person as the Comfort Coach and it just does not seem feasible that 1 person can be guaranteed to be available for all of these specifically timed interventions over a 1 year period. Budget for part of a back-up Comfort Coach would seem wise to assure maintaining the protocol intervention schedule.

Response #15:
Thank you for this excellent and important question. **Regarding percentage of effort, the University of Michigan Medical School recognizes mentoring effort of faculty up to 5% of**

their time concurrent with their ongoing research activities. Each mentor will serve in an advisory capacity as experts in their field, sharing guidance in their area of expertise within their mentoring time.

As PI, my individual effort and time will be provided through my T32 training grant. This grant, funded by the NIH, provides 100% protected time for research. The BCBS project will be the main focus of the upcoming year of training time and will include my effort in providing the necessary direction, team support, research design, and commitment to successfully complete the project. I am also supported by personnel and resources by virtue of my position as a research fellow at the Center for Healthcare Outcomes and Policy (CHOP) and the Institute for Healthcare Policy and Innovation (IHPI).

Additional details for the main investigators and mentors in this project and brief descriptions of their effort and role in this project are provided in Response #8.

The Reviewer's concern about the comfort coach role and workload is well-taken. Salary support for 100% effort of a comfort coach is the main budgeted expense in this study and the most important. This individual will function as the lead, coordinating comfort coach but will not be the sole individual available and trained to provide the required non-pharmacological comfort coach interventions. We are in the process of training a two-tiered team of 13 specialists to demonstrate competencies with providing comfort coach interventions in a program-wide effort to provide back-up coverage to the lead comfort coach. While obtaining full salary support for a main comfort coach is essential to successfully performing this trial, the supplementary support team will provide daytime back up and nighttime on-call assistance.

Another consultant on our project, Lindsay Heering, MS, CCLS, is the Administrative Director for the Department of Child and Family Life. **Ms. Heering coordinates and leads our group of specialists and has provided a letter of support demonstrating the institution-wide level of commitment to this project, including the already in-progress training the described team of specialists available around-the-clock, 24 hours per day, 7 days per week to supplement the role of the lead comfort coach.**

Comment #16: The second concern is the request for only .28 RA. This individual is expected to consent and administer surveys to all 154 subject plus screening of others who are excluded or choose not to participate. Additionally, there are data planned to be abstracted from charts for pain and opioid use while an inpatient. Time allocated does not appear adequate. These additional costs and costs related to qualitative portion of study could substantially increase overall budget for completion of this project.

Thank you again for the opportunity to review this project. I find it highly worthy of support with great potential to enhance patientcare with attention for both the effectiveness and efficiency of the intervention.

<u>Response #16</u>:
Thank you for bringing up this important point. This project will require substantial support, as outlined by the Reviewer. In addition to all of the investigators, mentors, and consultants on our project, **the Department of Cardiac Surgery is also invested in this project and its success. As discussed on our phone call, the budget contribution from BCBSM has been adjusted to $70,000. The balance exceeding $70,000 will be specifically to cover effort of support personnel for this project and will be provided by the Department through Dr. Deeb's limited discretionary research budget, as outlined in the REVISED budget. Most importantly, the Department of Cardiac Surgery is committed to providing a dedicated research coordinator in-kind for this study, with an estimated 10% total effort and benefits, which we have added in the revised budget. In addition to a research assistant and**

research coordinator as budgeted, the Office of Patient Experience has a dedicated graduate intern capable of focusing training efforts on this study and who will also be integrated into our team and fill a role similar to the research assistant. The research coordinator in conjunction with Dr. Deeb and myself will oversee all aspects of the study, recruit and supervise research assistants, communicate with the study team and consultants, and facilitate regular in-person progress meetings. In addition, the research coordinator will work with the main comfort coach to coordinate all required comfort coach interventions. The research coordinator, assistants, and comfort coach, will meet weekly with Dr. Deeb, Julie Piazza and myself to review the status and progress of the study. As a team we will anticipate the needs of the personnel covering the patients and schedule accordingly.

Reviewer #3

This is an admirably thorough plan to evaluate an innovative and potentially promising intervention in cardiovascular surgery. The team has carefully considered risks and opportunities and will evaluate patient behavior and acceptance, opioid use, and overall cost of care. Cardiac surgery patients represent a small but clinically important and costly patient population. The trial will detect only major shifts in results. It is viewed as preliminary; if successful, larger scale trials will be necessary to extend the findings beyond Michigan Medicine.

Comment #17: The intervention is carefully detailed. Patients in the experimental group will receive six specific coaching sessions. The coach will be one of the investigators. The coach has a number of tools at her disposal (described in an appendix this reviewer did not receive).

However, the intervention is the project's biggest weakness. "Hawthorne effect" is a clear risk. Expanding the intervention to multiple providers, even within Michigan Medicine, will require a clear understanding of the coach's actions and management of the coach/patient relationship. Success in this experiment does not guarantee extension.

The opportunity is much greater than the risk.

Response #17:
Thank you for making a number of important points. While Julie Piazza is a certified child life specialist and my main co-investigator, she currently serves as the Senior Project Manager in the Office of Patient Experience and will not be the 100% salary-support comfort coach for this project. As a certified child life specialist and the original pioneer of this trial from the inception of the feasibility trial, Julie is heavily invested in supporting the entire group in this project, but will not be personally delivering comfort coach interventions. The fully supported, dedicated comfort coach will function as the lead, coordinating comfort coach but will not be the sole individual available and trained to provide the required non-pharmacological comfort coach interventions. We are in the process of training a team of 13 child life specialists to demonstrate competencies with providing comfort coach interventions in a program-wide effort to provide back-up coverage to the lead comfort coach.

We agree that a potential limitation to this study is the risk of the Hawthorne effect, as well as internal spillover between nurses and other members of the care team. To help mitigate these risks, we will conduct educational in-service sessions with physicians, advanced-practice team members, and nurses to make this trial well-known to providers before it begins. With these sessions we aim to minimize the spillover of specific comfort coach techniques to other patients during the trial period. If due to the Hawthorne effect the patients included in our trial as subjects report increased satisfaction with a comfort coach versus those without one, we consider this finding noteworthy and

hope to further investigate reasons for effectiveness and ineffectiveness through our planned qualitative analysis.

To help mitigate risks identified by the Reviewer in regards to the intervention, **each comfort coach at the time of each intervention regularly documents the specific non-pharmacological interventions utilized by that patient, so that individualized techniques are recorded through a standard method, which should help inform and guide subsequent interventions. In addition to supporting Ms. Piazza in her role in this project, the Office of Patient Experience is also committed to helping ensure that this intervention, if efficacious, can be implemented more broadly in adult patient settings. We also recognize that success does not guarantee extension, but also agree the opportunity is much greater than the risk!**
